# Assessment of the Effectiveness and Cost-Effectiveness of Tailored Web- and Text-Based Smoking Cessation Support in Primary Care (iQuit in Practice II): Protocol for a Randomized Controlled Trial

**DOI:** 10.2196/17160

**Published:** 2020-07-14

**Authors:** Joanna Proctor, Felix Naughton, Melanie Sloan, Sarah Hopewell, James Brimicombe, A Toby Prevost, Edward C F Wilson, Tim Coleman, Stephen Sutton

**Affiliations:** 1 University of Cambridge Cambridge United Kingdom; 2 University of East Anglia Norwich United Kingdom; 3 Imperial College London London United Kingdom; 4 University of Nottingham Nottingham United Kingdom

**Keywords:** text messaging, smoking cessation, internet-based intervention, adults, smokers, tobacco, primary care

## Abstract

**Background:**

The prevalence of smoking is declining; however, it continues to be a major public health burden. In England, primary care is the health setting that provides smoking cessation support to most smokers. However, this setting has one of the lowest success rates. The iQuit in practice intervention (iQuit) is a tailored web-based and text message intervention developed for use in primary care consultations as an adjunct to routine smoking cessation support with the aim of increasing success rates. iQuit has demonstrated feasibility, acceptability, and potential effectiveness.

**Objective:**

This definitive trial aims to determine the effectiveness and cost-effectiveness of iQuit when used as an adjunct to the usual support provided to patients who wish to quit smoking, compared with usual care alone.

**Methods:**

The iQuit in Practice II trial is a two-arm, parallel-group, randomized controlled trial (RCT) with a 1:1 individual allocation comparing usual care (ie, pharmacotherapy combined with multisession behavioral support)—the control—with usual care plus iQuit—the intervention. Participants were recruited through primary care clinics and talked to a smoking cessation advisor. Participants were randomized during the initial consultation, and those allocated to the intervention group received a tailored advice report and 90 days of text messaging in addition to the standard support provided to all patients.

**Results:**

The primary outcome is self-reported prolonged abstinence biochemically verified using saliva cotinine at 6 months after the quit date. A sample size of 1700 participants, with 850 per arm, would yield 90% power to detect a 4.3% difference in validated quit rates between the groups at the two-sided 5% level of significance. The Cambridge East Research Ethics Committee approved the study in February 2016, and funding for the study was granted from May 2016. In total, 1671 participants were recruited between August 2016 and July 2019. Follow-up for all participants was completed in January 2020. Data analysis will begin in the summer of 2020.

**Conclusions:**

iQuit in Practice II is a definitive, pragmatic RCT assessing whether a digital intervention can augment the impact of routine smoking cessation support in primary care. Previous research has found good acceptability and feasibility for delivering iQuit among smoking cessation advisors working in primary care. If demonstrated to be cost-effective, iQuit could be delivered across primary care and other settings, such as community pharmacies. The potential benefit would likely be highest where less behavioral support is delivered.

**Trial Registration:**

International Standard Randomized Controlled Trial Number (ISRCTN): 44559004; http://www.isrctn.com /ISRCTN44559004.

**International Registered Report Identifier (IRRID):**

DERR1-10.2196/17160

## Introduction

### Public Health Burden of Smoking

In 2015, the total estimated smoking-related cost to the UK National Health Service (NHS) was GB £2.6 billion (US $3.3 billion), of which GB £1.1 billion (US $1.4 billion) was within primary care settings [1]. Smoking-related morbidity accounts for 489 300 hospital admissions and 77,800 deaths in England each year. About 14.4% of adults in England currently smoke [2], and despite the fact that smoking prevalence has fallen over recent years, this equates to approximately 6.4 million adults aged >20 years [3]. Prevalence has reduced mostly in the 18- to 24-year age group; however, the proportion of adults still smoking is highest among the unemployed and those of a lower economic status. There has been some improvement in the quit success rate of this population, which may be due to the European Union tobacco products directive, which was finalized in 2017 [4]. Prohibition of smoking in public spaces and the use of smoking cessation services may have contributed to the reduction in smoking prevalence over the last 15 years [5]. However, to achieve the Department of Health’s vision of a smoke-free generation by 2035 [6], there is a strong need to increase the effectiveness and cost-effectiveness of existing interventions embedded within health care in addition to other tobacco control initiatives.

### Primary Care as a Setting for Smoking Interventions

Since 2012, when revisions were made to the quality and outcomes framework’s reporting of smoking status, general practitioners (GPs) have been incentivized to offer and record stop-smoking support to all registered patients [7]. Therefore, primary care is an important setting for smoking cessation support. Physicians, nurses, and other health care practitioners are in a prime position to engage with and provide smoking cessation support to patients presenting at specialized health clinics (eg, asthma, hypertension, diabetes) and general consultations. Many practices train at least one health care practitioner to offer and deliver smoking cessation support, and the two most commonly trained practitioners to deliver support (ie, health care assistants and nurses) are equally effective at delivering this support [8].

The stop smoking services (SSS) follow the National Institute for Health and Care Excellence recommendations [9] to provide one-to-one behavioral support, discussing and providing pharmacotherapy for smokers wishing to quit. Although the number of people attending NHS stop smoking services in primary care and general practice has fallen in recent years [2,10], the proportion of successful quit attempts (self-report at 4 weeks after a quit attempt) remains steady at 51% [10]. A systematic review of 44 studies showed that patients receiving cessation support from nurses or other health care practitioners offering advice, counseling, and other strategies increased their chances of a successful quit attempt (at 6 months after quit date) by 29% (relative risk [RR] 1.29; 95% CI 1.21 to 1.38), 15.7% in the treatment arm (nursing intervention) versus 12.2% in the control arm (minimal intervention) [11]. In this review, *nursing intervention* was defined as the provision of any kind of support or advice by nurses, and the control group comprised those who saw the same nurse but received only brief advice and self-help materials. In addition, increasing the amount of behavioral support can further aid the smoker to quit (RR 1.15; 95% CI 1.08 to 1.22) [12].

### Text Messaging and Tailored Interventions for Smoking Cessation (Effectiveness and Cost-Effectiveness)

Tailored interventions use data collected on or about an individual to make the information provided to them more personally relevant, increasing the likelihood that it will be read, understood, and acted upon. Tailored self-help interventions can be more effective than nontailored materials (RR 1.28; 95% CI 1.18 to 1.37) [13] and are better than no help at all (RR 1.34; 95% CI 1.19 to 1.51) [13]. In addition, text messaging programs can increase quit rates (RR 1.54; 95% CI 1.19 to 2.00) [14].

Currently, 96% of adults in the United Kingdom have access to a mobile phone, and in 2017, 6.4 billion SMS text messages were sent [15]. A Cochrane review of 12 studies assessing the effectiveness of text-based mobile phone interventions to support a quit attempt found a positive effect of mobile phone interventions on abstinence compared with controls at 6 months (RR 1.67; 95% CI 1.46 to 1.90), 9.3% in the treatment arm versus 5.6% in the control arm [16]. The majority of interventions in these trials were SMS text message based, whereas the control arms varied from daily nontailored text messages to no intervention.

The York Health Economics Consortium [17] concluded that there was not enough evidence concerning the cost-effectiveness of NHS-related smoking interventions. Their review showed some cost benefits, but the studies analyzed were far-reaching and included smoking cessation support in hospital settings, some very old studies, and studies that were of both low- and high-intensity interventions.

However, smoking cessation interventions have been shown to be cost-effective in many populations, including low-income populations [18] and people with chronic obstructive pulmonary disease (COPD) [19]. Tailored smoking cessation interventions have also been shown to be cost-effective [20], with the cost benefit increasing the older the smoker is at the time of quitting (Table 1—data adapted from the txt2stop trial) [20]. However, although quitters in the txt2Stop trial had access to NHS SSS, the intervention was delivered as an adjunct to these services and not through them.

**Table 1:** A summary of incremental costs and quality-adjusted life years gained per 1000 participants.

Characteristics	Age (years)		
	<30	30-40	>40
Incremental cost, GB£ (US$)	−11,066 (−13,682)	−30,320 (−37,487)	−74,214 (−91,756)
Incremental quality-adjusted life years	20	27	38

The intervention in this trial (iQuit in practice intervention [iQuit]) is a tailored smoking cessation system designed for use by health care practitioners during the delivery of routine cessation support. iQuit is the culmination of research into the effectiveness of tailored smoking cessation advice reports [21-24] and evidence from trials using SMS text messages as a means of delivering tailored messages to smokers to assist them in their quit attempt [20,25-31]. Using a web-based questionnaire, the practitioner asks smokers a series of questions that are used to tailor an advice report. iQuit also delivers a 90-day program of automated, tailored, and interactive text messages, designed to support the smoker in their quit attempt.

### The iQuit in Practice Pilot Trial

iQuit has previously been assessed for feasibility, acceptability, and short-term effectiveness in a randomized controlled trial (RCT) [32]. In the trial, we found good acceptability for iQuit from participants and advisors. A total of 93.7% of participants found the texts easy to understand, 67.7% felt receiving support by text message acceptable, and 44.8% said that the text messages had helped them to quit smoking. It took advisors approximately 8 min (mean 7.7 min SD 4.0) to deliver the intervention and gave a mean score of 4.6 (SD 0.7, 5-point scale) for ease of using the web-based questionnaire within a consultation [32]. In terms of its effect on short-term abstinence (Table 2), the primary focus of the trial, we found no significant between-group differences. However, although not prespecified, we found statistically significant between-group differences for both self-reported 6-month prolonged abstinence at the 6-month follow-up and for continuous abstinence.

**Table 2:** Summary of smoking outcomes from the iQuit in Practice pilot trial.

Smoking outcomes for the iQuit in Practice pilot trial		Control arm, n (%)	Intervention arm, n (%)	Absolute difference, % (95% CI)	Odds ratio (95% CI)
**Primary outcome**					
	Self-reported 2-week point-prevalence abstinence at 8-week follow-up	122 (40.3)	135 (45.2)	4.9 (−3.0 to 12.7)	1.22 (0.88 to 1.69)
**Secondary outcomes**					
	Carbon monoxide–verified 2-week point-prevalence abstinence at 4-week follow-up after quit date	71 (23)	81 (27)	3.7 (−3.3 to 10.6)	1.21 (0.84 to 1.76)
	Self-reported 3-month prolonged abstinence at 6-month follow-up	70 (23)	76 (25)	2.3 (−4.5 to 9.1)	1.13 (0.78 to 1.65)
**Additional outcomes**					
	Self-reported 6-month prolonged abstinence at 6-month follow-up	27 (9)	45 (15)	6.1 (0.9 to 11.4)	1.81 (1.09 to 3.01)
	Continuous abstinence (4-week, 8-week, and 6-month follow-ups)	19 (6)	34 (11)	5.1 (0.6 to 9.8)	1.92 (1.07 to 3.45)

The RR for the long-term intervention effect at 6 months was 1.69 (95% CI 1.08 to 2.65). The estimated probability that the intervention would produce a small intervention effect equivalent to an RR of at least 1.2 is 93%, and the probability of a medium effect size of 1.5 is 70%.

This trial is therefore a definitive, pragmatic RCT in primary care to assess the effectiveness and cost-effectiveness of iQuit on biochemically validated abstinence at a 6-month follow-up. It will be assessed against the *usual practice* for smoking cessation consultations.

### Trial Objectives

The study aims to assess the effectiveness and cost-effectiveness of iQuit when delivered alongside usual care compared with usual care alone.

### Design

iQuit in Practice II is a two-arm, parallel-group RCT with 1:1 individual allocation comparing usual care (control) with usual care plus iQuit (intervention).

## Methods

### Practices: Recruitment

Participants were recruited from general practices in the East of England under the remit of the Eastern and North Thames clinical research networks. Our pilot study [32] suggested that 22 is a feasible minimum target of participating smokers per practice; therefore, we set out to approach 66 practices over 36 months to achieve our initial proposed sample size of 1452 participants. However, participant recruitment was monitored and reported monthly to the study team by the trial coordinator. Detailed graphs and charts of overall and per practice activity informed whether more or fewer general practices were needed to achieve the target.

General practices were eligible if they had at least one smoking cessation advisor trained to deliver level 2 smoking cessation advice (or were willing to be trained to that level). Practices were not to be participating in any other smoking cessation research studies, and all smoking cessation advisors had to have internet access from a computer in their consultation room and access to a printer. Training and site initiation took place in a single 2-hour session. Training included informed consent, using the iQuit web-based questionnaire, and an explanation of RCTs.

### Participants: Inclusion and Exclusion Criteria

Patients were eligible for the study if they met all of the following criteria: (1) a current tobacco smoker (ie, they usually smoked at least one cigarette a day and had smoked in the past 7 days), (2) able to read and understand English and could provide written informed consent, (3) ≥18 years, (4) had a mobile phone and were familiar with sending and receiving text messages, (5) willing to participate in the study and follow study procedures, and (6) not enrolled in another formal smoking cessation study or treatment program at the time.

Health care practitioners were prompted to check whether patients met the eligibility criteria when they signed on to the iQuit in Practice computer program. The ability to read and understand English was a subjective decision by the practitioner based on their knowledge of the patient and the patient’s medical records.

Patients were excluded if they were considered by their GP to be unsuitable for the study for any reason. There was no proforma of ineligible conditions. It was at the discretion of the GP and health care practitioner to decide whether or not the patient should take part in the study. GPs were asked to screen lists of known smokers before invitation letters were sent. Reasons for noninclusion included mental illness, terminal illness, or dementia. Patients with existing medical conditions such as COPD or heart disease were not excluded unless their GP considered them unsuitable. As individual allocation worked effectively in the pilot trial, with no contamination found [32], patients were also not excluded if more than one person per household was participating in the trial.

Participants were not re-enrolled into the trial if they had not been successful in their quit attempt. However, this did not exclude them from receiving further support from their smoking cessation advisor.

### Participants: Recruitment

**Figure 1:**
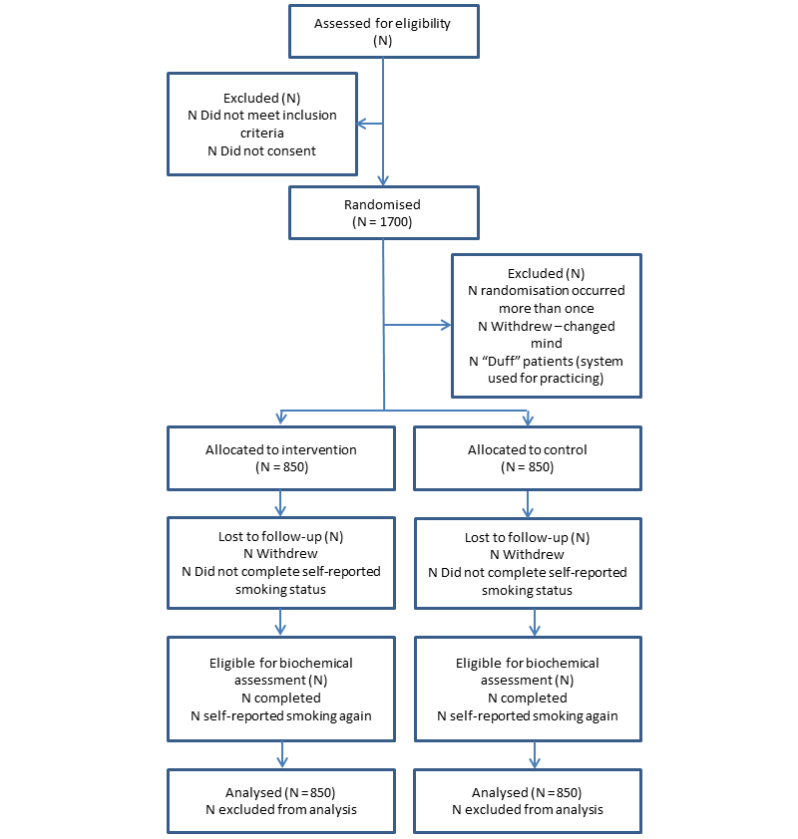
Projected trial profile.

Participants were recruited using 3 methods (Figure 1 shows the projected trial profile):

1. Opportunistic recruitment: All health care practitioners in participating practices were encouraged to identify potential participants. Smokers presenting at other clinics (eg, health checks, asthma clinics, COPD clinics) and expressing a desire to quit smoking were advised to make an appointment with the smoking cessation advisor.
2. Self-referral: Patients could refer themselves for smoking cessation support.
3. Proactive recruitment: Practices were encouraged to mail study invitations to smokers identified from their database. In addition to a covering letter, patients received an information sheet and were encouraged to make an appointment with the smoking cessation advisor at the surgery.

Participant recruitment in GP practices was monitored (as previously described), and a number of strategies were used to ensure the sample size was achieved (eg, regular newsletters [sometimes tailored to the practice]). Further support and training for underperforming practices was available. Practices were also encouraged to use national promotions (eg, Stoptober and national no smoking day) to encourage recruitment. Posters and leaflets were available for practices to display in their waiting rooms.

### Interventions

#### Control

In-person SSS support, which is mainly delivered one to one, is usual practice for smoking cessation consultations in primary care. During the first session, the advisor discusses with the patient the reasons for why they smoke and why they wish to quit. They also assess the patient’s level of nicotine dependence using validated tools such as the Fagerstrom test for nicotine dependency, the heaviness of smoking index, or the Urge to Smoke Questionnaire. They discuss past quit attempts and support the patient to set a quit date. To aid the quit attempt, patients are offered pharmacotherapy: varenicline, buproprion, or nicotine replacement therapy (NRT) products (eg, patches, gum, lozenges, inhalators, mouth, and nasal sprays). The patient’s expired-air carbon monoxide (CO) level is also measured using the practice’s own Smokerlyzer. Follow-up appointments by the smoking cessation advisor are offered at 1, 2, 3, and 4 weeks after the quit date to monitor progress and to offer advice on handling withdrawal symptoms and difficult situations, to monitor CO levels, and to ensure that the patient has an adequate supply of medication [33].

All participants in our trial received this level of support (*usual care*).

#### Intervention

In addition to the *usual care* described earlier, participants allocated to the intervention arm received the following.

#### Tailored Advice Report

iQuit uses participant answers to the iQuit web-based questionnaire to instantly generate a highly tailored detailed advice report (Multimedia Appendix 1), approximately 4 A4 pages in length. The program is informed by (1) theories of smoking cessation and behavior change, including social cognitive theory [34] and the perspectives of change model [35]; (2) findings from previous studies; (3) feedback from the iQuit in Practice I trial [32]; and (4) best practice guidance from the National Centre for Smoking Cessation Training [36]. To ensure adequate tailoring, iQuit asks detailed questions about an individual’s smoking habits and history, including nicotine dependence, motivation and determination to quit, reasons for quitting, self-image, pros and cons of quitting, perceived difficult situations, children, living with other smokers, social support, and current health problems. The advice report contains detailed advice on quitting tailored to 25 items from the web-based questionnaire. The program can generate over 3300 million different reports.

#### Tailored Text Messages

A 90-day program of automatically generated tailored text messages (Multimedia Appendix 2) is sent to the participant’s mobile phone, beginning the day before their quit date. Participants receive either 0, 1, or 2 messages each day (approximately 1.2 on average), with fewer messages toward the end of the 90 days. The messages are a further refinement of those developed for the version evaluated in the first trial with some additional features [32]. Texts were amended to reflect the current trend in SMS messaging (eg, the return of full length rather than abbreviated words, ie, text instead of txt). However, content, style, and frequency remained unchanged, as 64.1% of participants in the pilot trial found them useful and 93.7% of participants found them easy to understand. The texts are designed to remind participants about their quit attempt, provide information about reasons for quitting, increase and maintain motivation, boost confidence at quitting, and provide coping strategies for difficult situations. Messages are individually tailored using baseline information collected through the web-based questionnaire with additional information obtained via interactive messages sent to participants at 4, 5, and 8 weeks during the program. The 4- and 8-week messages ask about current smoking status (ie, have they smoked in the last week. Participants respond by texting *Y* or *Yes*, *N* or *No* and those that have smoked in the last week are invited to text in a new quit date). The 5-week message asks participants about their confidence in quitting for good using a 5-point scale and provides feedback in response. Participants can text *STOP*, email, or telephone the study team at any time to stop receiving further messages. For added distraction, participants can text *QUIZ* to receive a general knowledge quiz question. After submitting their answer, they receive a text telling them whether their answer is correct or incorrect. If they do not respond, they will automatically receive the correct answer after approximately 5 min. Participants can also text *HELP* if they are tempted to smoke, or *SLIP* if they have had a lapse to receive further support. If participants want to increase or decrease the frequency of the text messages, they can text *MORE* or *LESS*.

### Sample Size, Power, and Precision

In the iQuit in Practice I trial, the quit rates (self-reported prolonged abstinence at 6 months) were 8.9% and 15.1% in the control and intervention groups, respectively [32]. If we assume a 90% response rate to biochemical verification and that 90% of these are confirmed as nonsmokers (as advised by the chair of the trial steering committee, Dr Jamie Brown from University College London), this gives the estimated validated quit rates of 7.1% and 12.1% for control and intervention, respectively. An absolute increase of 5% quitting would be a worthwhile and scalable effect for this low-cost intervention. Detecting an effect of this size with 90% power using a two-sided chi-square test at the 5% significance level requires 726 participants per arm (nonresponders at follow-up assumed to be smoking), and 1452 in total.

In the first 12 months of recruitment, the response rate to biochemical validation was lower than expected. The observed response rates suggested that we would fall short of the 282 biochemically validated nonsmokers required to maintain 90% power (column 3 of Table 3 gives further details). On the basis that, we needed the same number (n=282) of validated nonsmokers. Using the observed response rates of participants to biochemical validation (64%), a revised sample size of 1700 participants was proposed. With 850 participants per arm, the study has 90% power to detect a 4.3% difference in validated quit rates of 10.3% versus 6.0% at the two-sided 5% level of significance. The trial steering committee (TSC) and the research ethics committee approved the revised sample size of 1700 participants.

**Table 3:** Sample size assumptions.

Raw numbers	Scenario 1, n	Scenario 2, n	Scenario 3, n
Sample size	1452	1452	1700
Self-reported abstinence at 6 months postquit date	348	479	561
Response to biochemical validation	314	307	287
Validated nonsmoker	282	245	287

### Randomization

Randomization was stratified by smoking cessation advisor, so that each advisor would see approximately equal numbers of intervention and control participants. The allocation sequence is generated by a computer-based random number generator using random permuted blocks with block sizes of 4 and 6 to make the sequence difficult to predict, while avoiding a major imbalance between intervention and control groups if a block is incomplete at the end of recruitment. The sequence is stored on the web server database, accessible only to the data manager and the chief investigator. Allocation is made after the questions in part 1 of the web-based iQuit program have been completed.

### Procedure: Initial Consultation

At the start of the initial appointment, the smoking cessation advisor logs onto the web-based iQuit program, talks through the information sheet (Multimedia Appendix 3) and goes through the set of 6 eligibility questions with the patient. If eligible, the advisor obtains written consent (Multimedia Appendix 4) from the patient following good clinical practice guidelines [37].

Once informed consent has been obtained, participants complete a short health utility questionnaire (EQ-5D-5L) [38] in which they report any problems with mobility, self-care, usual activities, pain, anxiety, and depression. Following this, the smoking cessation advisor continues with *usual care* before returning to the iQuit program. There are 2 parts to the iQuit program. Part 1 includes questions on gender, age, cigarette consumption, the longest period of abstinence, strength of motivation, and determination to quit smoking. A quit date (which must be within 2 weeks of the consultation) is also recorded in this section along with the CO reading taken earlier in the consultation. It is only after these questions have been completed that the computer randomly allocates the participant to either the control or the intervention group (Multimedia Appendix 5).

For participants allocated to the control group, there are no further questions. They are reassured that even though they are in the control group, their contribution to the research study is still valuable. They are also reminded that the research team will follow them up in 6 months. The smoking cessation advisor finishes the appointment in the usual way, making clinical follow-up appointments with the participant wherever possible.

For participants randomized to the intervention group, there is a further set of questions (part 2). These questions include the tailoring questions described in the intervention section earlier. Finally, the participant’s mobile phone number is entered, and their preferred way of being referred to in the text messages. At the end of part 2, the smoking cessation advisor prints the generated advice report and hands it to the participant to take away and read. Intervention participants start receiving the 90-day program of text message support beginning the day before their quit date.

### Procedure: Follow-Up

All participants in the control and intervention groups are followed up 6 months after their initial quit date. The follow-up comprises 3 parts:

1. A question to ascertain the success of their quit attempt: Participants are asked to respond to the question “have you smoked at all since your quit attempt” with either A1, A2, or A3 meaning that they have had no cigarettes, not more than five cigarettes in total or smoked more than five cigarettes in total, respectively.
2. A saliva sample for biochemical validation: Participants who respond to the first question with A1 or A2 also receive a saliva sampling kit for biochemical validation (cotinine and anabasine in saliva are important biomarkers for determining a participant’s smoking status) [39,40].
3. A more detailed questionnaire (Multimedia Appendix 6) that participants can complete on the web, on a printed copy, or over the telephone.

The preferred method for asking about smoking status is indirectly through either a text message or via email. However, if neither of these contact details were available, but the participant had given a landline telephone number, a researcher, blind to group allocation, called the participant to ask the question over the phone. Participants who had not provided a telephone number or an email address were sent the detailed questionnaire, which included the primary outcome question, through the post.

Participants who responded to the first question by text or email were sent a link to complete the questionnaire on the web. Participants who responded to the question over the telephone were given the option of completing the questionnaire at that time, were sent the questionnaire by post, or completed it on the web. All participants who answered with either A1 or A2 to the initial question were sent a saliva sampling kit through the post for biochemical validation of their quit attempt.

All participants were followed up with a telephone call (where possible) if there was a missing follow-up component (ie, the saliva sample has not been received by the laboratory within 14 days of it being sent and the questionnaire has not been returned). Where it was not possible to reach the participant using these methods, a further sample kit and the questionnaire was sent to the address we held.

If there was no response to any form of communication within 90 days of their quit date, participants were categorized as lost to follow-up and treated as smokers for the statistical analysis.

### Data Collection, Management, and Analysis

All personal data are stored on a secure server within the University of Cambridge Clinical School computing services. Access to the trial database is through a 2-factor authentication system (Signify). All data are anonymized before analysis and publication.

Baseline data were collected through the iQuit program, and follow-up data were collected as described earlier. Saliva samples were sent to ABS laboratories (BioPark). Cotinine levels of <15 ng/mL suggest abstinence from smoking by the participant [41]. Cotinine, however, does not distinguish between nicotine obtained through tobacco smoke and nicotine obtained through NRT. Therefore, a second assay to measure anabasine is run if the cotinine concentration is >15 ng/mL.

### Data Monitoring

We have not appointed a data monitoring committee as this is a low-risk trial assessing a behavioral intervention. However, a TSC has been formed and meets to oversee recruitment and follow-up and to provide comments and expertise on the statistical analysis plan and significant protocol changes.

## Results

In February 2016, the Cambridge East Research Ethics and the Health Research Authority approved the study to begin recruiting. Funding began in May 2016 and between August 2016 and July 2019, 1671 participants were recruited. Follow-up was completed by January 2020 and data collection will commence in the summer of 2020.

The working protocol for the study is version 7, dated March 27, 2018 (Multimedia Appendices 7 and 8)

### Outcomes

In line with the Russell Standard, the primary outcome is self-reported prolonged abstinence over the entire 6-month follow-up period (allowing for up to five cigarettes in total), combined with 7-day point prevalence with biochemical verification at 6 months [40]. In the event that a participant was unwilling to provide a saliva sample, they could provide a CO reading instead. CO readings were taken at their physician’s surgery (Multimedia Appendices 9 and 10).

Secondary outcomes include (1) CO-verified abstinence at the 4-week quit date follow-up for at least 2 weeks, assessed by the smoking cessation advisor; (2) self-reported prolonged abstinence over the whole of the 6-month follow-up period (allowing for up to five cigarettes in total); (3) self-reported 7-day point-prevalence abstinence at the 6-month follow-up; and (4) cost and utility measures (ie, the time required to complete the iQuit program and resource use, including use of cessation medication).

Process measures, all assessed from the follow-up questionnaire include (1) the number of serious quit attempts lasting ≥24 hours during the 6-month follow-up period, (2) motivation and confidence in quitting, (3) use of strategies to help avoid smoking or lapsing during the 6-month follow-up period, and (4) (intervention group only) evaluation of the tailored advice report and text messaging program (eg, how helpful they found them, how they felt about the number of texts sent).

### Statistical Analysis

A detailed statistical analysis plan was completed by the senior trial statistician dated August 16, 2018, and approved by the TSC. The final analysis will be performed after all the follow-up data has been collected; there is no interim analysis. The statistician will be blind to group allocation until data queries have been resolved, and the statistical analysis plan has been followed. The analysis will take an intention-to-treat (ITT) strategy approach. The ITT population comprises all patients that have been randomized, regardless of finding any participant later to have been ineligible or any controls who have mistakenly received the behavioral intervention, or any intervention arm participants who had not received the behavioral intervention. A per-protocol analysis is not planned because the number of participants not receiving an intervention was very low in our previous study [32]. The focus will be on reporting 95% CIs for estimates of effect size. Statistical tests will be two-tailed and assessed at the 5% significance level. Tests will be used sparingly and restricted largely to addressing stated hypotheses as detailed in the statistical analysis plan, so reporting of *P* values will be limited.

### Primary Effectiveness Analysis

The primary analysis of the primary outcome will involve obtaining a point estimate of the intervention effect as the difference in proportions, with 95% CI and the corresponding *P* value for the intervention effect from the Pearson chi-square test. The primary analysis and sensitivity analysis for handling missing primary outcome data (primarily assuming this as *not abstinent*) will together comprise the ITT strategy. The detailed statistical analysis plan describes the secondary reporting of relative effects, such as odds ratio and RR, which should be useful for comparison with other studies and meta-analyses. The statistical analysis plan describes further methods for this sensitivity analysis and for exploratory secondary analysis of the primary outcome, indicated by unexpected covariate imbalance. The statistical analysis plan provides further information as to why, a priori, the primary analysis is decided to be unadjusted for the stratifier (smoking cessation advisor), given the low numbers of primary outcome events per stratifier categories.

Subgroup variables that will be examined as potential moderators of the intervention are medication use (categorized into NRT or varenicline), nicotine dependence (based on the heaviness of smoking index, categories of 7+ and <7), and the Index of Multiple Deprivation (5 quintile-based categories). The approach to subgroup analysis will involve assessing the significance of the interaction of a subgroup variable with arm allocation and summarizing the intervention effect with a 95% CI within each category of the subgroup variable.

### Secondary Effectiveness Analyses

The key secondary effectiveness outcomes are binary, and a Pearson chi-square test will be used unless assumptions are unexpectedly contraindicated. Ordinal response outcomes (and their changes from baseline, where collected) will be evaluated between arms using methods for continuous outcomes, in consideration of the large sample size, unless categories are few and sparse, requiring categorical analysis methods outlined further in the statistical analysis plan. Where the baseline is available, analysis of covariance will be used for continuous outcomes. Alternatively, the Student *t* test will be used. If there is evidence of differential variance between arms (eg, Levene F-ratio), then an alternative test based on an appropriate modification to fractional degrees of freedom will be reported if the variance ratio is large enough to materially alter the result and conclusion.

### Economic Analyses

The economic evaluation will estimate the incremental cost per incremental individual quitting smoking at 6 months (defined as self-report with biochemical verification: the primary outcome measure) and per incremental quality-adjusted life year (QALY) gained over 6 months.

The setting of the study is English primary care (and is assumed to be generalizable to the United Kingdom as a whole). The analytic perspective is that of the NHS over a time horizon of 6 months. No interim analyses are planned. Costs and outcomes will not be discounted as the follow-up period is less than one year. Cost items comprise the index consultation with the smoking cessation advisor (data derived from the iQuit program), text messages, prescription medications, and other primary care contacts. QALYs will be calculated from the EQ-5D-5L using the recommended set of utility weights at the time of analysis.

### Process Evaluation

Many process measures fall into either ordinal or binary categories. Those considered to be potential mediators include motivation, confidence in quitting, use of cognitive behavioral lapse prevention strategies, medication use, and use of additional NHS cessation support. Process measures that are intervention-arm-only are also ordinal or binary, and will be summarized descriptively in terms of averages and percentages, and include reading advice reports, reading advice texts, finding them helpful, and viewing messages (annoying versus pleasing).

For continuous process measures, to obtain valid CIs, the nonparametric bootstrap method will be considered for use when the distribution is highly skewed, and an absolute difference presentation and interpretation is important.

For process measures proximal to the behavior, such as motivation and confidence in quitting, chi-square trend tests for between-arm evaluation will be reported after assumption checking.

For measures proximal to the intervention, such as use of strategies to help avoid smoking or lapsing during the follow-up period of the individual’s experience in the trial, Pearson chi-square test, chi-square trend test, Student *t* test will primarily be used depending on response distribution and distributional assumptions, and interpretability of point and ideally interval estimate provision.

## Discussion

The purpose of this trial is to evaluate the effectiveness and cost-effectiveness of digital adjunctive support to usual smoking cessation care in primary care. The trial has been designed following the Standard Protocol Items: Recommendations for Interventional Trials [42] and Consolidated Standards of Reporting Trials [43,44] guidelines (Multimedia Appendix 11), and feedback from the pilot trial [32] to maximize both internal and external validities.

### Trial Design

A cluster-randomized design was considered for the trial but decided against. The randomization built into the web-based iQuit computer program is easily implemented, maintains allocation concealment, and avoids selection bias. In addition, if there is a clustering of outcomes, an individually randomized design requires a smaller sample size to detect an effect. The computer program randomizes participants during the initial consultation. The allocation sequence is not accessible to smoking cessation advisors or participants in advance, and the advisors and participants remain blinded to group allocation until after the first set of questions.

However, there are disadvantages to individual randomization in trials of behavioral interventions. Participants may be disappointed to learn that they will not be receiving the iQuit intervention and feel they are not active participants in the trial.

This may, in part, be due to a lack of understanding of RCTs and the purpose of control groups. Therefore, in the participant information sheet and with smoking cessation advisors in training, we provide an explanation of RCTs and why people allocated to a control group are as important to research as the group receiving the intervention. Advisors also reassure participants they will still receive support from them during their quit attempt.

However, there is the potential for this to lead to systematic differences between intervention and control conditions in the usual care component as smoking cessation advisors try to compensate for this disappointment. However, participants not receiving the intervention still have access to all available resources in the same way as the group receiving the intervention. Therefore, any additional support given to the control arm would attenuate any intervention effect and thus have a conservative effect on the results.

### Recruitment

We encouraged GP practices to recruit opportunistically rather than proactively. Although proactive invitation letters can act as a prompt for participation, this approach was not very successful during our pilot trial [32] and would likely be insufficient to recruit the numbers we require. However, opportunistic recruitment has some disadvantages: (1) more time is needed to talk with potential participants about the study, which in a busy general practice can be a challenge, and (2) recruiting participants who have had to make a quick decision about taking part in the study. We therefore encouraged GP practices to hand out study information to patients attending other consultations (eg, diabetes clinics) and in the waiting room to maximize the time available for reading information and coming to a decision. With increased use of electronic-cigarettes [2], smokers seeking support from smoking cessation services have fallen over recent years [6]. Therefore, recruitment was monitored internally and various strategies used to encourage practices to be more active in their recruitment. Quarterly newsletters of study progression were produced, and regular contact with smoking cessation advisors enabled us to work with them to identify individual issues with recruitment. In the majority of cases, the over-riding problem was a fall in numbers seeking support through the surgery. Therefore, further GP practices were recruited as necessary to ensure we maintained a steady recruitment rate to reach our target.

### Outcome Assessment

The preferred method for obtaining the initial response is by text or email. Every attempt is made to obtain self-reported smoking status in this way. However, where there has been no response to initial attempts, participants are called instead. The interviewer is initially blind to the participant’s group allocation; however, during the course of the telephone interview, they will become unblinded as details of the participants’ quit attempts are discussed. Self-report outcomes, however, are naturally subject to bias in whichever way they are collected. We will conform to the Russell Standard definitions of abstinence, and a saliva sample will be obtained from all participants who self-report a successful quit attempt to determine their smoking status. This presents further disadvantages; for example, it may yield a lower than expected response rate. However, attrition is monitored along with recruitment, and decisions will be made to compensate for a lower than anticipated return of saliva samples. For example, early on during the trial, we decided to increase the sample size to compensate for a lower than expected return.

### External Validity

In this trial, external validity is increased by using smoking cessation advisors, employed by practices, rather than research staff to deliver the intervention and the control intervention. We used minimal inclusion and exclusion criteria for practices and participants, and iQuit was designed to be easily incorporated into usual care, whereas the text messages were delivered independently of the practice. Opportunistic recruitment also had the potential to include smokers who would not necessarily have replied to a study intervention, thereby increasing the diversity of the study population. However, there still remains a limitation in the study design, as smokers with a poor grasp of written English may struggle with the advice report and texts.

### Potential Benefits of the iQuit Program

If the iQuit program is demonstrated to be cost-effective, it could be delivered across primary care in all regions. Over 100,000 smokers set a quit date in the NHS each year, of which over 38,000 [45] are seen in primary care. It has the potential to reach a large number of smokers seeking support to quit. It may also be beneficial for smokers receiving cessation support in other settings, such as a pharmacy, which treats over 20,000 smokers per year. The potential benefit would likely be highest where less behavioral support is delivered.

## Appendix

Multimedia Appendix 1 iQuit example advice report.

Multimedia Appendix 2 iQuit in Practice example text messages version 1.0, December 16,2015.

Multimedia Appendix 3 iQuit in Practice participant information sheet version 6.0, May 24, 2018.

Multimedia Appendix 4 iQuit in practice participant consent form version 3, December 06,2016.

Multimedia Appendix 5 Schedule of enrolment and interventions for the iQuit study.

Multimedia Appendix 6 iQuit in Practice intervention group 6-month follow-up questionnaire version 5, September 28, 2018.

Multimedia Appendix 7 iQuit in Practice protocol v7.0_March 27, 2018.

Multimedia Appendix 8 iQuit in Practice peer review comments.

Multimedia Appendix 9 Baseline variables.

Multimedia Appendix 10 Outcome variables.

Multimedia Appendix 11 Consolidated Standards of Reporting Trials e-HEALTH checklist.

## Abbreviations

CO: carbon monoxide
COPD: chronic obstructive pulmonary disease
CRN: clinical research network
GCP: good clinical practice
GP: general practitioner
iQuit: iQuit in practice intervention
ITT: intention to treat
NHS: National Health Service
NRT: nicotine replacement therapy
PIS: participant information sheet
PPI: patient and public involvement
QALY: quality-adjusted life year
RCT: randomized controlled trial
REC: research ethics committee
RR: relative risk
SCA: smoking cessation advisor
SSS: stop smoking service
TSC: trial steering committee
