# Supplementary material for: Assessment of the Effectiveness and Cost-Effectiveness of Tailored Web- and Text-Based Smoking Cessation Support in Primary Care (iQuit in Practice II): Protocol for a Randomized Controlled Trial
Source: JMIR Res Protoc. 2020 Jul 14;9(7):e17160. doi: 10.2196/17160 (PMC7388034; doi:10.2196/17160)
Supplement: Multimedia Appendix 1 [file resprot_v9i7e17160_app1.doc]

**Example Advice Report**

**Quit Smoking Advice Report for Sharon Mckenna**

You have made the decision to quit. Congratulations! This is a positive step and you deserve to feel proud of yourself. This report is intended to help you personally, based on your answers to the questionnaire. We hope you find it useful, and that it will help you to become aware of your thoughts and attitudes towards your smoking. We hope that it will also increase your confidence by offering you skills to help you to cope when tempted to smoke.

You set a quit date for the 16th of October. Well done. Setting a date to carry out your intention shows your commitment to quitting, and reinforces your specific intention to quit. Your motivation and determination to quit for good are high. These qualities are important for successful quitting, and show that you are prepared to put effort into quitting to get results. Now reaffirm your decision, make sure you are fully prepared to carry out your plan and commit yourself to going through with it.

## Your reasons for quitting

Your main reason for quitting is because of pressure from others. You may see this as nagging, because your smoking annoys them. But the pressure to quit from your friends and family is probably because they are concerned for your health. You are important to them, and they don't want to see you suffer because of your habit.

You are probably aware of the major health risks of smoking, including those that particularly apply to women, such as infertility. Although you may not have a health problem yet, you can't afford to be complacent, because you may already have unseen damage. Quitting smoking is the single most important change you can make to reverse any damage. When you stop smoking, you will experience some improvements to your health immediately. These will continue and your risk of severe health problems will decline.

Your children will also benefit when you quit. Passive smoking can cause smoking related diseases, as well as being a risk factor in childhood asthma, bronchitis and cot death. Older children will also have the benefit of a positive role model if they are tempted to take up smoking. Children are more likely to smoke if they see parents smoking.

But don't forget also the amount of money you can save by quitting. You said that you smoke 30 cigarettes per day, which must cost you about £63 per week. By quitting, in one year you would save £3285. Think what you could do with this money, and with improved health you will be able to enjoy your treats more.

## Your carbon monoxide level

Carbon monoxide (CO) is one the most harmful elements of cigarette smoke, and is absorbed into your blood when you smoke. CO from cigarette smoke joins onto the red blood cells, reducing their ability to carry oxygen to the heart; this increases your chances of a heart attack or stroke. Your CO level is 20ppm (parts per million), cutting the oxygen carrying power of your blood by between 3% and 4%. But by stopping smoking your CO level will immediately drop to below 4ppm. Your blood will carry more oxygen, and you will have better circulation and more energy.

## Changing your self-image

You see yourself as addicted to smoking and we understand that you must feel that quitting is a very difficult task. But these thoughts are just messages from your brain telling you that you need nicotine. Confront these thoughts and tell yourself that you can overcome them and you don't need the nicotine. It is the act and routine of smoking that is part of your life. Challenge your beliefs about the value of smoking, learn to think of yourself separately from the habit, and imagine life as a non-smoker. Keep calling yourself a non-smoker, practise saying it until it becomes part of you. If you have previously tried to quit, don't be discouraged. Learn from your past experiences and plan new ways of coping next time.

At your current level of smoking there are products that you can use which will increase your chances of success, and we recommend that you use one of these. Your Practice Nurse may already have arranged a prescription for you. The medication will help you through the early days while you are breaking free of the habit. Be determined and stick with it, and the craving will diminish over time.

## Quitting is a positive step

You are worried about gaining weight if you stop smoking. We understand this concern, this is something that many quitters worry about. While you may be tempted to eat to replace smoking, you don't have to eat things that will make you gain weight. Try to replace the habit with healthy snacks and with other, healthier activities. If you approach quitting as a positive move, achieving control over the habit and improving your life, you will feel good about yourself and will also be able to control your weight. The average weight gain is less than 7lb, and remember that a weight gain this small is still preferable to and less harmful to your health than continuing to smoke.

## For when you may be tempted

Your responses indicate that you find it most difficult to resist when you are feeling angry or stressed. This is not unusual, dealing with temptations when you are stressed is difficult, and many people use cigarettes to help them to cope with these negative emotions. But smoking is just a distraction, there are other distractions and ways of coping. Think carefully about the situations where you used to smoke. Think about your feelings in those situations, and think about how it will feel to not smoke in those situations, whether smoking will make the problem go away.

There is no one way to deal with these urges, you need to try different ways to see what works for you. Plan ahead for these stressful times and develop a range of strategies to cope with them. Practise dealing with negative feelings without smoking. This will increase your confidence that you can do without.

Here are some tips to try:

- Ask yourself 'What would a cigarette do for me right now?' Remind yourself that cigarettes don't really help you with your problems, you can deal with them another way.
- Relax, do some deep breathing
- Take a walk, do some exercises.
- Replace cigarettes with something else e.g. gum
- Leave the situation for a few minutes to distance yourself from the source of the stress.
- Focus on another task.
- Do something, anything, to take your mind off it.
- Think about consequences of having a cigarette on your resolve to quit.
- Reward yourself and give yourself small treats that you enjoy with the money that you are saving.

Think about times when you are confident and you can resist smoking. Make use of this confidence, and use it when you feel the need to smoke.

## Don't go it alone

You live with other people who don't smoke, who will support you in your attempts to quit. That's great! Make the most of this support from your family and friends, ask for their encouragement and tell them that you appreciate them listening and helping at a difficult time. However, when you are out, avoid smokers who might demotivate you, and don't rely entirely on the support of friends, quitting is up to you, it will be your success, not theirs.

Finally, congratulations on your decision to quit. This is one of the best decisions of your life. Remember to plan ahead and use the skills you have been practising. You have many more resources than you are consciously aware of. Stay focused on your task, and maintain a positive frame of mind, and it will help you in your resolve. Remember there is nothing to be lost from quitting smoking, but everything to gain.

Good Luck! Remember: You Can Do It
