# Supplementary material for: Assessment of the Effectiveness and Cost-Effectiveness of Tailored Web- and Text-Based Smoking Cessation Support in Primary Care (iQuit in Practice II): Protocol for a Randomized Controlled Trial
Source: JMIR Res Protoc. 2020 Jul 14;9(7):e17160. doi: 10.2196/17160 (PMC7388034; doi:10.2196/17160)
Supplement: Multimedia Appendix 2 [file resprot_v9i7e17160_app2.docx]

iQuit in Practice, example text messages

“Hi Sarah, welcome to iQuit in Practice, a personal program of quitting support. We hope you enjoy it and that it helps in your quit attempt. The iQuit team.”

“As well as removing cigarettes and ashtrays, ask any visitors not to smoke inside. Now's a great time to freshen up the curtains and wipe away any smoke stains.”

“Quitting is great for your skin. Several studies have found measureable improvements in the skin tone of quitters, often within in the first four weeks!”

“By staying quit, your immune system will start to recover, meaning you'll soon be better able to fight off colds and other illnesses.”

“After all your effort to get this far, make sure you reward yourself. It's a real achievement, and this time it's for good. :-)”

“Don't believe 'just one' is okay: A 2010 review found that even 'light' smokers were at much more risk of lung problems and cataracts compared to non-smokers.”

“Hi Sarah, great job so far. Just make sure you don't give yourself any excuses. You decided to quit because you wanted to QUIT.”
