# Supplementary material for: Assessment of the Effectiveness and Cost-Effectiveness of Tailored Web- and Text-Based Smoking Cessation Support in Primary Care (iQuit in Practice II): Protocol for a Randomized Controlled Trial
Source: JMIR Res Protoc. 2020 Jul 14;9(7):e17160. doi: 10.2196/17160 (PMC7388034; doi:10.2196/17160)
Supplement: Multimedia Appendix 3 [file resprot_v9i7e17160_app3.doc]

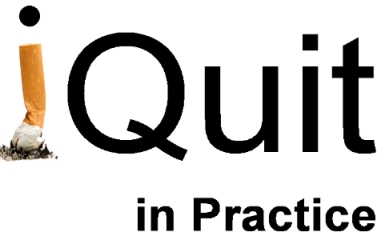


# Appendix 5.iQIP PIS

**Participant Information Sheet**

**Version 6.0**

**Dated 24/05/2018**

You are being invited to take part in the iQuit in Practice study.

**What is the purpose of the research?**

Researchers in the Primary Care Unit at the University of Cambridge have developed a stop smoking intervention called iQuit, which provides a tailored advice leaflet and a 90-day program of tailored text messages designed to support people in their quit-attempt.

To help you decide whether or not to take part, please read the following leaflet carefully and discuss it with others if you wish. We are happy to answer any questions and our contact details can be found at the bottom of this page.

This study aims to assess the effectiveness of iQuit when used alongside smoking cessation support provided at GP practices (‘usual care’). To do this, we need to compare a group of participants who receive usual care with a group who receive usual care plus the iQuit intervention.

**Why have I been invited?**

You have been invited to take part because according to your general practice records you are a smoker and are aged over 18 years.

**Do I have to take part?**

No, it is your decision and your healthcare will not be affected either way. If you do decide to take part, you can withdraw at any time without giving a reason. If you decide not to participate, you need not do anything further.

###### Further information

For any further information regarding the study, please contact the iQuit in Practice study co-ordination team:

Telephone: 01223 761760

Email: [iQuit@medschl.cam.ac.uk](mailto:iQuit@medschl.cam.ac.uk)

Address: iQuit in Practice Research Study. Primary Care Unit

Institute of Public Health, University of Cambridge

Forvie Site, Robinson Way

Cambridge, CB2 0SR

Office hours: 9.00am – 5.00pm

**What will happen to me if I take part in the study?**

If you would like to take part, please make an appointment to see the smoking cessation advisor (SCA) in your GP practice. The appointment will take about 30 minutes, during which the SCA will explain the study, check your eligibility, and ask you to sign a study consent form.

Once you have given consent, the SCA will conduct routine smoking cessation support which includes advice about quitting, using a Smokerlyser to measure the carbon monoxide (CO) level in your breath, and asking you to set a quit date within the next 14 days. The SCA might also arrange a voucher for nicotine replacement therapy or other medication to help you to quit. It is likely that you will also be invited for a follow-up appointment or contact call at 4-weeks to see how you got on with your quit attempt.

After completing your consultation, the SCA will ask you to complete a short questionnaire about your health (EQ-5D). S/he will access the iQuit computer program and, with your help, complete a short online questionnaire. S/he will ask about your age, your cigarette consumption and your motivation to quit. The SCA will also enter your carbon monoxide reading taken earlier in the consultation The computer will then allocate you into either the control group (usual care), or the intervention group (usual care + iQuit intervention). The allocation is random, so you have a 50% chance of being in the control group, and a 50% chance of being in the intervention group.

Control group

If you are allocated to the control group, the SCA will conclude your appointment, and any follow-up appointments will occur as usual.

Intervention group

If you are allocated to the intervention group, the SCA will ask a few more questions about your motivation to quit and situations when you might find it difficult. The iQuit program will use your questionnaire responses to produce a personalised quitting advice report which the SCA will print for you to keep. They will then conclude your appointment and conduct any follow-up appointments as usual.

As well as the advice report, you will receive a 90-day program of text messages beginning the day before your quit date. You will be sent 0, 1 or 2 messages on each day, which will be automatically generated and tailored to you based on your answers to the iQuit questionnaire. You will also be able to text ‘HELP’ for an instant message if you are tempted to smoke, or ‘SLIP’ for a message if have had a lapse. You can text ‘STOP’ at any time to stop receiving messages. **Texts are charged at the standard rate but free if included in your operator’s text bundle**. Alternatively, if you would prefer not to text, you can email or telephone the study team to request that messages are stopped. At the end of the 90 day program, no further text messages will be sent. The text message intervention will not be offered post-study.

**Follow-up** **(all participants)**

Six months after your first consultation with the SCA, a member of the study team will contact you by telephone and/or email or post to ask you some brief questions about your smoking status, health and study experiences. Whether you are in the control or intervention group, your feedback is **equally** important. If you inform us that you have stopped smoking we would like to collect a saliva sample from you. This will enable our research to meet the criteria set out in a document called the Russell Standard, a standard for measuring smoking abstinence that enables results from different studies to be meaningfully compared. We will post you a saliva sample kit, instructions for collecting a sample, and freepost envelope addressed to the analysis lab: ABS Laboratories, Welwyn Garden City, Hertfordshire. The saliva sample will be stored in freezers at -20 ^o^ until they are analysed after which they will destroy in accordance with “the Human Tissue Act (HTA). Any participant who does not wish to provide a saliva sample will be offered a home visit to provide a CO reading if preferred.

In addition to obtaining the above information from you at 6 months, we will also contact your GP surgery to ask for details about your 4-week quit outcome, and to obtain your ethnicity and occupational code.

**What are the possible risks of taking part?**

We do not foresee any risks from participating in this study. However, should you find any aspect distressing, we will take this seriously. If anyone in the intervention group finds any messages upsetting, we advise texting ‘STOP’ immediately (or email/telephone the study team) to end the messages, and contact the study team who will do their best to resolve the issue.

**What are the possible benefits of taking part?**

Whether you are allocated to the intervention group or the control group, you will receive advice and support that may help you to quit smoking and you will contribute to testing an approach that may help others to quit in the future.

###### What happens to the information collected from you?

Information obtained throughout the study will remain strictly confidential. All data will be stored securely in accordance with the Data Protection Act and the University of Cambridge policy on data security. Electronic data will be stored on a secure partition on a University of Cambridge file server.

Cambridge University is the sponsor for this study based in the United Kingdom. We will be using information from you and your medical records in order to undertake this study and will act as the data controller for this. This means that we are responsible for looking after your information and using it properly. Cambridge University will keep identifiable information about you for 6 months after the study has finished.

Your rights to access, change or move your information are limited, as we need to manage your information in specific ways in order for the research to be reliable and accurate. If you withdraw from the study, we will keep the information about you that we have already obtained. To safeguard your rights, we will use the minimum personally-identifiable information possible.

You can find out more about how we use your information at <https://www.medschl.cam.ac.uk/research/privacy-notice-how-we-use-your-research-data/>

Your doctor’s surgery will collect information from you and your medical records for this research study in accordance with our instructions.

Cambridge University will use your name and contact details to contact you about the research study and to make sure that relevant information about the study is recorded for your care, and to oversee the quality of the study. Individuals from Cambridge University and regulatory organisations may look at your medical and research records to check the accuracy of the research study. Your doctor’s surgery will pass these details to Cambridge University along with the information collected from you and your medical records. The only people in Cambridge University who will have access to information that identifies you will be people who need to contact you to find out how you got on in your quit attempt or audit the data collection process. The people who analyse the information will not be able to identify you and will not be able to find out your name or contact details.

Cambridge University will keep identifiable information about you for 6 months after the study has finished.

The following organisations will also have access to your data:

1. The text messages will be transferred by a company called FastSMS. If you are in the intervention group, the company will have access to your name (your first name will be used in some of the messages), your mobile phone number and the content of the message. The company privacy policy states that all information will remain confidential and will not be disclosed to any third parties.
2. A data processing company might be used to process anonymised data from questionnaire responses. The questionnaires will not contain any identifiable data.
3. ABS Laboratories; however, the saliva sample you send to the laboratory will include only your study ID.
4. It is possible that once the results from the study have been published, researchers external to the university including outside the EU will request access to the study data. This would only happen at least three years after the study has finished (April 2023). Such requests will be considered by the study team however only data that has had all identifiable personal information removed will be made available.

After the study has ended, anonymised data may be stored securely for up to 20 years, to ensure that it has been properly reported. Secure storage will be in accordance with national guidance.

###### What will happen to the results of the research study?

After completing the study, you will be offered a summary of the results. The results will be published in scientific journals and presented at conferences. Anonymised raw data will also be made available through open source. In every instance, data will be anonymised, and you will not be identifiable in any report or publication.

**What if there is a problem?**

If you have a concern about any aspect of this study, please contact Dr Felix Naughton who will do his best to address the issue in consultation with the Chief Investigator, Professor Stephen Sutton if necessary. If you wish to speak to someone outside the study team, please contact Jonathan Mant (jm677@medschl.cam.ac.uk), Professor of Primary Care Research and head of the Primary Care Unit. In the event of a complaint about your NHS care, please contact your GP, who will advise you on the appropriate procedure.

**What should I do next?**

If you would like to take part, please contact your GP practice to arrange a consultation with the smoking cessation advisor. If you are later unable to attend, please contact the surgery to reschedule.

###### Who is organising and funding the research?

The study is being conducted by the Primary Care Unit, University of Cambridge in collaboration with GP practices in the East of England. This study is funded by Cancer Research UK. Indemnity insurance, which also covers the design of the study at all participating sites, and the management and conduct of the study at University of Cambridge sites, is provided by the University’s insurers Newline.

All research in the NHS is looked at by an independent Research Ethics Committee to protect your safety, rights, wellbeing and dignity. This study has been reviewed and given favourable opinion by NRES Committee East of England, Cambridge East (16/EE/0030).

**Thank you for taking the time to read this information leaflet**

**Statements that you will be asked to agree to on the consent form if you decide you would like to take part**

| - I confirm that I have had the opportunity to read the participant information sheet for the above study (version x, dated xx/xx/xxxx), and have had any questions answered satisfactorily. |
| --- |
|  |
| - I understand that my participation in the study is completely voluntary, and that I am free to withdraw at any time without giving a reason and without my medical care being affected. |
|  |
| - I am aware that all information I provide will remain confidential and that anonymised data may be entered into a database by a professional data processing company. |
|  |
| - I agree to my data being shared with researchers external to the University including outside the EU and understand that any information that can identify me will have been removed beforehand. |
|  |
| - I understand that monitors and auditors from the funder, sponsor, NHS Trust and regulatory inspectors may require access to the data to check that that the study is being properly conducted. |
|  |
| - I agree to my mobile phone number being used in this study. |
|  |
| - I understand that I will be asked to provide a saliva sample at six months if I have successful stopped smoking. |
|  |
| - I agree for the study team to obtain details of my ethnicity, occupation and 4-week quit outcome from my GP practice. |
|  |
| - I agree to take part in the above study. |
