# Supplementary material for: Assessment of the Effectiveness and Cost-Effectiveness of Tailored Web- and Text-Based Smoking Cessation Support in Primary Care (iQuit in Practice II): Protocol for a Randomized Controlled Trial
Source: JMIR Res Protoc. 2020 Jul 14;9(7):e17160. doi: 10.2196/17160 (PMC7388034; doi:10.2196/17160)
Supplement: Multimedia Appendix 4 [file resprot_v9i7e17160_app4.doc]

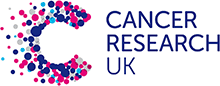


##


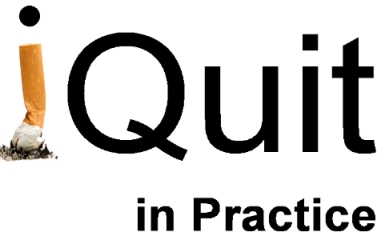


**Department of Public Health and**

**Primary Care**

Institute of Public Health

Forvie Site

Robinson Way

Cambridge

CB2 0SR

**Nurse I.D.**

**Participant I.D.**

# iQuit in Practice: A Randomised Controlled Trial to assess the effectiveness of iQuit in supporting smokers to quit smoking.

**CONSENT FORM** *please initial*

*each box*

| - I confirm that I have had the opportunity to read the participant information sheet for the above study (version 5, dated 04.01.2017), and have had any questions answered satisfactorily. |  | *please initial* |
| --- | --- | --- |
|  |  |  |
| - I understand that my participation in the study is completely voluntary, and that I am free to withdraw at any time without giving a reason and without my medical care being affected |  | *please initial* |
|  |  |  |
| - I am aware that all information I provide will remain confidential and that anonymised data may be entered into a database by a professional data processing company. |  | *please initial* |
|  |  |  |
| - I agree to my data being shared with researchers external to the University including outside the EU and understand that any information that can identify me will have been removed beforehand. |  | *please initial* |
|  |  |  |
| - I understand that monitors and auditors from the funder, sponsor, NHS Trust and regulatory inspectors may require access to the data to check that that the study is being properly conducted. |  | *please initial* |
|  |  |  |
| - I agree to my mobile phone number being used in this study. |  | *please initial* |
|  |  |  |
| - I understand that I will be asked to provide a saliva sample at six months if I have successful stopped smoking. |  | *please initial* |
|  |  |  |
| - I agree for the study team to obtain details of my ethnicity, occupation and 4-week quit outcome from my GP practice. |  | *please initial* |
|  |  |  |
| - I agree to take part in the above study. |  | *please initial* |

| Name (participant) – *please print*  *Title / first name / surname* | | | | **Today’s** Date *(dd/mm/yy)* | | | | | | | | Signature: | | | | | | | | | | | | | | | | | | |
| --- | --- | --- | --- | --- | --- | --- | --- | --- | --- | --- | --- | --- | --- | --- | --- | --- | --- | --- | --- | --- | --- | --- | --- | --- | --- | --- | --- | --- | --- | --- |
| Address (participant) | | | | | | | | | | | | | | | | | | | | | | | | | | | | | | |
| *include full*  *postcode here* | | | | | | | | | | | | | |  | |  | | |  | |  | | |  | |  | | |  | |
| Email address: | Mobile number: | | | | | | | | | | | | | Home number: | | | | | | | | | | | | | | | | |
|  |  |  | |  |  |  |  |  |  |  | |  |  |  | |  |  | |  | |  |  | |  | |  |  | |  |
| Name of person taking consent – *please print* | | | | **Today’s** Date *(dd/mm/yy)* | | | | | | | | | | Signature of person taking consent: | | | | | | | | | | | | | | | | |

* 3 copies to be retained- top (white) to research team, middle (yellow) to practice medical records, bottom (pink) to the participant.
