# Supplementary material for: Assessment of the Effectiveness and Cost-Effectiveness of Tailored Web- and Text-Based Smoking Cessation Support in Primary Care (iQuit in Practice II): Protocol for a Randomized Controlled Trial
Source: JMIR Res Protoc. 2020 Jul 14;9(7):e17160. doi: 10.2196/17160 (PMC7388034; doi:10.2196/17160)
Supplement: Multimedia Appendix 5 [file resprot_v9i7e17160_app5.docx]

**Figure 2.** Schedule of enrolment and interventions for the iQuit study.

| Time point | Enrolment | Allocation | Post allocation | | | |
| --- | --- | --- | --- | --- | --- | --- |
|  | *−t_1_* | 0 | *t*_1_ (iQuit text start) | *t*_2_ (4-week visit to smoking advisor) | *t*_3_ (iQuit texts *stop*) | *t*_4_ (follow-up) |
|  | | | | | | |
| **Enrolment** | | | | | | |
| Eligibility screen | X^a^ | ^b^— | — | — | — | — |
| Informed consent | X | — | — | — | — | — |
| EQ5D^c^ questionnaire | — | X | — | — | — | — |
| iQuit web-based questionnaire | — | X | — | — | — | — |
| Allocation | — | X | — | — | — | — |
| **Interventions** | | | | | | |
| iQuit intervention | — | — | ^d^ | X |  | X |
| Control group | — | — | — | X |  | X |

^a^X denotes treatment given at the indicated timepoint

^b^dashes denote no treatment given at the indicated timepoint

^c^Euroqol health utility questionnaire (EQ5D):

^d^straight line denotes the length of time the participant in the intervention group receives the intervention.
