# Supplementary material for: Assessment of the Effectiveness and Cost-Effectiveness of Tailored Web- and Text-Based Smoking Cessation Support in Primary Care (iQuit in Practice II): Protocol for a Randomized Controlled Trial
Source: JMIR Res Protoc. 2020 Jul 14;9(7):e17160. doi: 10.2196/17160 (PMC7388034; doi:10.2196/17160)
Supplement: Multimedia Appendix 6 [file resprot_v9i7e17160_app6.doc]

**
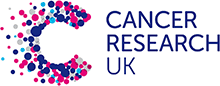
**


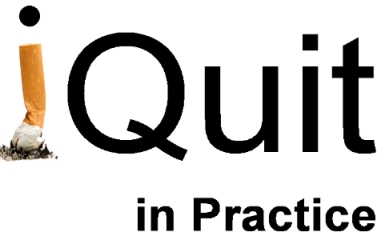


**Department of Public Health and**

**Primary Care**

**«PID3»**

**«PID5»**

**«NID4»**

**«NID3»**

**«NID2»**

**«NID1»**

Participants I.D. Nurse I.D.

**iQuit in Practice: 6 Month Follow Up Questionnaire**

Thank you for taking part in this study. We value your feedback and any comments which will help us to further develop the iQuit in Practice program for smokers in primary care.

- The questionnaire consists of 4 parts
  - PART 1 is about your current smoking behaviour
  - PART 2 is about the resources you may have used to assist you in your quit attempt.
  - PART 3 is about the report and text messages you received
  - PART 4 is about your current health.
- Please complete all the questions using a **black** pen only.
- Please answer all the questions providing as much information as possible.
- Your responses will be kept anonymous
- This questionnaire will take approximately 15 minutes to complete.
- Please return the completed questionnaire to the research team in the FREEPOST envelope provided

Please insert **today’s** date here:

D

D

M

M

Y

Y

**Would you like us to send you a copy of the results for the iQuit in Practice study?**

Yes No

**Would you be happy to speak to an interviewer about your experiences of the iQuit program and your quit attempt?**

Yes No

**Part 1: The following questions are about your** **current smoking behaviour**

Please answer question 1 even if you have already answered it by text or email

1. **Have you smoked since your initial quit date 6 months ago?**

No

Yes, between 1 and 5 cigarettes in total

Yes, more than 5 cigarettes in total

1. **Have you smoked in the last 7 days?**

Yes No

1. **Since your initial quit attempt after you set a quit date with your advisor approximately 6 months ago, how many additional serious quit attempts, lasting at least 24 hours, have you deliberately made?**
2. **How much do you want to quit for good?**

Not at all slightly moderately very much extremely

1. **How confident are you that you can quit smoking for good?**

Not at all slightly moderately very much extremely

**Part 2: We would like to know what resources and techniques you used (if any) to help you with your quit attempt**

1. **Are you currently or have you used any of the following medications or nicotine products to help you to stay quit in the last six months? (tick all that apply and provide dates. If you can’t remember exact dates, please write your best guess)**

Currently

using

Have used in the past six months

When did you start using? When did you stop?

Champix

/ /

/ /

/ /

/ /

/ /

/ /

/ /

/ /

/ /

/ /

/ /

/ /

/ /

/ /

Zyban

Nicotine patches

Nicotine gum/lozenges

Nicotine nasal/mouth spray

Nicotine inhalator

E-cigarettes

Other

/ /

/ /

(*Please specify*________________________________________________

_____________________________________________________________

None of the above

1. **Are you currently receiving or have you received advice or help with stopping smoking from any of the following in the last six months?**

**Please *do not* include your *initial* smoking cessation appointment with your advisor, but please *do* include any follow-up appointments, and any other contacts with other health professionals about stopping smoking *(tick all that apply and provide further information where asked).***

Practice nurse/healthcare assistant: How many visits in the last 6 months?

GP: How many visits in the last 6 months?

Pharmacist: How many visits in the last 6 months?

NHS stop smoking service: How many contacts in the last 6 months?

NHS helpline (telephone advice): How many calls in the last 6 months?

Other telephone helpline: How many calls in the last 6 months?

Internet:

Smartphone app:

Other (*Please specify):* ___________________________________________

None of the above

1. **Since you joined the study how many times have you tried the following** **to help you avoid smoking?**

|  | **Not used at all** | **Used 1-5 times** | **Used 6-10 times** | **Used more than 10 times** |
| --- | --- | --- | --- | --- |
| Focusing on other tasks | **□** | **□** | **□** | **□** |
| Avoiding spending time with other smokers | **□** | **□** | **□** | **□** |
| Changing my routine | **□** | **□** | **□** | **□** |
| Avoiding places where I would usually smoke | **□** | **□** | **□** | **□** |
| Avoiding stressful situations | **□** | **□** | **□** | **□** |
| Avoiding places where I can buy cigarettes | **□** | **□** | **□** | **□** |
| Trying not to think about smoking | **□** | **□** | **□** | **□** |

1. **Since you joined the study how many times have you tried the following** **to help you not smoke when you experienced a craving/urge?**

|  | **Not used at all** | **Used 1-5 times** | **Used 6-10 times** | **Used more than 10 times** |
| --- | --- | --- | --- | --- |
| Going for a walk or doing some exercise | **□** | **□** | **□** | **□** |
| Keeping my mouth busy e.g. chewing mints or gum | **□** | **□** | **□** | **□** |
| Contacting a friend/family member for support or distraction | **□** | **□** | **□** | **□** |
| Replacing smoking with something else e.g. food or drink | **□** | **□** | **□** | **□** |
| Using self-talk e.g. “I can do it” | **□** | **□** | **□** | **□** |
| Delaying smoking e.g. waiting a few minutes for the urge to pass | **□** | **□** | **□** | **□** |
| Using deep breathing | **□** | **□** | **□** | **□** |
| Reminding myself how bad I will feel if I smoke | **□** | **□** | **□** | **□** |
| Thinking about the harmful effects of smoking | **□** | **□** | **□** | **□** |
| Thinking about the benefits of quitting | **□** | **□** | **□** | **□** |
| Thinking about rewards I could have for not smoking | **□** | **□** | **□** | **□** |
| Doing things that help me relax | **□** | **□** | **□** | **□** |
| Thinking about saving money | **□** | **□** | **□** | **□** |
| Looking back at the study text messages I received | **□** | **□** | **□** | **□** |

**Part 3: The following questions are about the advice report and text messaging program**

1. **Did you read the advice report?**

| I read all of it more than once | I read all of it once | I read most  of it | I read some  of it | I did not read any of it | I did not receive an advice report |
| --- | --- | --- | --- | --- | --- |

1. **Did you read the text messages?**

| I read all of them, some more than once | I read all of them once | I read most of them | I read some of them | I did not read any of them | I did not receive any text messages |
| --- | --- | --- | --- | --- | --- |

1. **How helpful did you find the advice report? (Please tick the box that applies to you)**

| Extremely unhelpful | quite unhelpful | neither unhelpful  nor helpful | quite helpful | extremely helpful |
| --- | --- | --- | --- | --- |

1. **How helpful did you find the text messages? (Please tick the box that applies to you)**

| Extremely unhelpful | quite unhelpful | neither unhelpful  nor helpful | quite helpful | extremely helpful |
| --- | --- | --- | --- | --- |

1. **How did you find the text messages? (Please tick the box that applies to you)**

| Extremely annoying | quite annoying | neither annoying  nor pleasing | quite pleasing | extremely pleasing |
| --- | --- | --- | --- | --- |

1. **How did you feel about the number of texts sent?**

| Too many | About right | Too few |
| --- | --- | --- |

1. What did you think of the duration of the text messaging program overall (i.e. 12 weeks)?

| Too long | About right | Not long enough |
| --- | --- | --- |

1. **Please use this space to add anything else that you would like to say about the text messaging or advice leaflet that you received.**

*
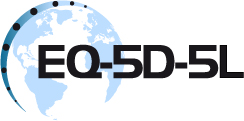
*

|  |
| --- |
| **Part 4: The Health Questionnaire** |
|  |
|  |
| **English version for the UK** |

*2009 EuroQol Group EQ-5D™ is a trade mark of the EuroQol Group UK (English) ©*

| Under each heading, please tick the **ONE** box that best describes your health **TODAY**. | |
| --- | --- |
| MOBILITY |  |
| I have no problems in walking about | ❑ |
| I have slight problems in walking about | ❑ |
| I have moderate problems in walking about | ❑ |
| I have severe problems in walking about | ❑ |
| I am unable to walk about | ❑ |
| SELF-CARE |  |
| I have no problems washing or dressing myself | ❑ |
| I have slight problems washing or dressing myself | ❑ |
| I have moderate problems washing or dressing myself | ❑ |
| I have severe problems washing or dressing myself | ❑ |
| I am unable to wash or dress myself | ❑ |
| USUAL ACTIVITIES *(e.g. work, study, housework, family or leisure activities)* |  |
| I have no problems doing my usual activities | ❑ |
| I have slight problems doing my usual activities | ❑ |
| I have moderate problems doing my usual activities | ❑ |
| I have severe problems doing my usual activities | ❑ |
| I am unable to do my usual activities | ❑ |
| PAIN / DISCOMFORT |  |
| I have no pain or discomfort | ❑ |
| I have slight pain or discomfort | ❑ |
| I have moderate pain or discomfort | ❑ |
| I have severe pain or discomfort | ❑ |
| I have extreme pain or discomfort | ❑ |
| ANXIETY / DEPRESSION |  |
| I am not anxious or depressed | ❑ |
| I am slightly anxious or depressed | ❑ |
| I am moderately anxious or depressed | ❑ |
| I am severely anxious or depressed | ❑ |
| I am extremely anxious or depressed | ❑ |

10

0

20

30

40

50

60

80

70

90

100

5

15

25

35

45

55

75

65

85

95

| - We would like to know how good or bad your health is **TODAY**. |
| --- |
| - This scale is numbered from 0 to 100. |
| - 100 means the best health you can imagine. 0 means the worst health you can imagine. |
| - Mark an X on the scale to indicate how your health is **TODAY.** |
| - Now, please write the number you marked on the scale in the box below. |

The best health you can imagine

YOUR HEALTH TODAY =

The worst health you can imagine

**Thank you for completing this questionnaire**

**Please check you have completed all questions and post it to the research team in the FREEPOST envelope provided**
