# Supplementary material for: Assessment of the Effectiveness and Cost-Effectiveness of Tailored Web- and Text-Based Smoking Cessation Support in Primary Care (iQuit in Practice II): Protocol for a Randomized Controlled Trial
Source: JMIR Res Protoc. 2020 Jul 14;9(7):e17160. doi: 10.2196/17160 (PMC7388034; doi:10.2196/17160)
Supplement: Multimedia Appendix 7 [file resprot_v9i7e17160_app7.doc]

| 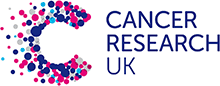 |  |  |
| --- | --- | --- |

| 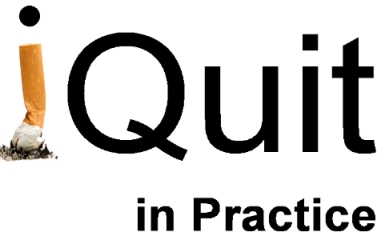  in Practice | Improving quit rates among smokers in primary care: Pragmatic trial of effectiveness and cost-effectiveness of a tailored web- and text message-based intervention for smoking cessation |
| --- | --- |
|  | STUDY PROTOCOL  Version number: 7.0  Version date: 27.03.2018 |

Table of Contents

[1 Protocol version control 3](#__RefHeading___Toc473645636)

[2 Study Contacts 4](#__RefHeading___Toc473645637)

[3 Study Synopsis 5](#__RefHeading___Toc473645638)

[4 Introduction 7](#__RefHeading___Toc473645639)

[5 Background 7](#__RefHeading___Toc473645640)

[6 The iQuit in Practice definitive trial 8](#__RefHeading___Toc473645641)

[6.1 Study objective: 8](#__RefHeading___Toc473645642)

[6.2 Study design: 8](#__RefHeading___Toc473645643)

[6.3 Trial intervention 8](#__RefHeading___Toc473645644)

[6.4 Sample size and power calculations 9](#__RefHeading___Toc473645645)

[6.5 Recruitment 9](#__RefHeading___Toc473645646)

[6.5.1 Practice recruitment 9](#__RefHeading___Toc473645647)

[6.5.2 Participant recruitment 10](#__RefHeading___Toc473645648)

[6.5.3 Recruitment procedure 10](#__RefHeading___Toc473645649)

[6.6 Informed Consent 10](#__RefHeading___Toc473645650)

[6.7 Consultation with the Smoking Cessation Advisor following consent to participate 11](#__RefHeading___Toc473645651)

[6.8 Frequency and duration of participant follow-up 11](#__RefHeading___Toc473645652)

[6.9 Confidentiality 12](#__RefHeading___Toc473645653)

[6.10 Measures 12](#__RefHeading___Toc473645654)

[6.11 Statistical analysis 13](#__RefHeading___Toc473645655)

[6.12 Time and event schedule for participants 14](#__RefHeading___Toc473645656)

[7 Trial Management and Governance Arrangements 15](#__RefHeading___Toc473645657)

[7.1 Research Team 15](#__RefHeading___Toc473645658)

[7.2 Sponsor 15](#__RefHeading___Toc473645659)

[7.3 Study Agreements 15](#__RefHeading___Toc473645660)

[7.4 Study Funding and NHS Cost implications 15](#__RefHeading___Toc473645661)

[7.5 Adoption of ‘iQuit in Practice’ onto the UKCRN Clinical Research Portfolio 15](#__RefHeading___Toc473645662)

[7.6 Insurance/Indemnity 15](#__RefHeading___Toc473645663)

[7.7 Adverse Incident reporting 16](#__RefHeading___Toc473645664)

[7.8 Intellectual Property (IP) 16](#__RefHeading___Toc473645665)

[7.9 Trial Steering Committee. 16](#__RefHeading___Toc473645666)

[7.10 Research & Development and Research Ethics Committee (REC) approvals 16](#__RefHeading___Toc473645667)

[7.11 Patient and Public Involvement 17](#__RefHeading___Toc473645668)

[7.12 Dissemination of research results 17](#__RefHeading___Toc473645669)

[7.13 Proposed study timetable 18](#__RefHeading___Toc473645670)

[Appendix 1: QUIZ questions 20](#__RefHeading___Toc473645671)

# Protocol version control

| Version number | Version Date | Protocol approvals and dates (e.g. REC etc) | Supersedes approved version number/date |
| --- | --- | --- | --- |
| 1.0 | 16/12/2015 |  |  |
| 3.0 | 05/02/2016 | To address issues raised by the HRA. | 1.0 (16/12/2015) |
| 4.0 | 14/04/2016 | Modifications made to the online questionnaire.  Eligibility age changed from 16-75 to 16 and over.  Follow-up measures to include collection of ethnicity, 4-week quit outcome and occupation from GP practices | 3.0 (05/02/2016) |
| 5.1 | 31/01/2017 | Information on data sharing to researchers outside the study team has been included.  Amendments to the follow-up procedure and outcomes for extra clarity  Updated the document with new information  Description of text messaging program now includes QUIZ; general knowledge quiz questions for when the participant needs to be distracted.  Corrected a typographical error in the age inclusion criteria from 16 to 18. | 4.0 (14/04/2016) |
| 6.0 | 12/12/2017 | To include details of another method of collecting primary outcome data | 5.1 (31.01.2017) |
| 7.0 | 27.03.2018 | To increase the sample size from1452 to 1700 | 6.0 (12/12/2017) |

# Study Contacts

For further information about the ‘iQuit in Practice study’, please contact:

| Chief Investigator: | Professor Stephen Sutton  Professor of Behavioural Science  Primary Care Unit  Dept. Public Health & Primary Care  University of Cambridge  Institute of Public Health  Forvie Site, Robinson Way  Cambridge, CB2 0SR  Email: [srs34@medschl.cam.ac.uk](mailto:srs34@medschl.cam.ac.uk)  Tel: 01223 330594 |
| --- | --- |
| iQuit Research Team | Email: iQuit@medschl.cam.ac.uk  Mobile: 01223 760761  Secure fax: 01223 762540 |

# Study Synopsis

| **Study short title** | iQuit in Practice | |
| --- | --- | --- |
| **Study full title** | Improving quit rates among smokers in primary care: Pragmatic trial of effectiveness and cost-effectiveness of a tailored web- and text message-based intervention for smoking cessation | |
| **Chief investigator** | Professor Stephen Sutton | |
| **Chief investigator’s employing Institution** | University of Cambridge | |
| **Sponsor** | University of Cambridge | |
| **Funder** | Cancer Research UK | |
| **Grant ref** | C1345/A20005 | |
| **ISRCTN** | ISRCTN44559004 | |
| **UKCRN ID** | 30934 | |
| **Study Duration** | 4 years (01/05/2016 to 30/04/2020) | |
| **Setting** | Primary Care: up to 66 NHS general practice surgeries, primarily in the East of England, UK. | |
| **Participants** | Current Smokers, aged 18+ | |
| **Design** | Two parallel group randomised controlled trial (RCT) | |
| **Randomisation** | 1:1 randomisation, at the level of the participant. | |
| **Sample size** | Total 1700 (850 in each group) | |
| **Treatment groups** | Two groups to be compared: | |
|  | **Control Group:** | Participants receive ‘usual care’ for smoking cessation |
|  | **Intervention Group:** | Participants receive ‘usual care’ for smoking cessation PLUS a printed patient-tailored advice report generated by web-based software, followed by a 90-day program of patient-tailored SMS text messages |
| **Primary Outcome measure** | The primary outcome measure is self-reported prolonged abstinence over the whole 6-month follow-up period (allowing for up to five cigarettes in total), combined with 7-day point prevalence with biochemical verification at 6 months that conforms to the Russell Standard. | |
| **Secondary Outcome measure(s)** | Secondary outcome measures include:   1. CO-verified abstinence at 4-week follow-up from quit date for at least 2 weeks, assessed by the smoking cessation advisor (SCA). 2. Self-reported prolonged abstinence over the whole six months follow-up period (allowing for up to five cigarettes in total) 3. Self-reported 7 day point-prevalence abstinence at 6-month follow-up.   (d) Cost and utility measures: duration of iQuit questionnaire completion; resource use (including use of cessation medication); EQ-5D. | |
| **Follow-up** | 1. Text/email/questionnaire/telephone call at six months by researcher to establish smoking status 2. Biochemical verification packs posted to those claiming prolonged abstinence | |
| **Analysis** | The two groups will be compared using χ2 tests and logistic regression analysis. Results will be reported in terms of absolute difference in percentages or proportions and the odds ratio, with 95% confidence intervals. Analysis will be intention-to-treat, where all those randomised are analysed, and those lost to follow-up assumed to be smoking. Sensitivity analyses will be conducted using a range of less severe assumptions including a complete-case analysis (Jackson et al, 2014) | |

# Introduction

‘iQuit in Practice’ is an intervention developed by researchers at the University of Cambridge to improve abstinence rates in those accessing stop smoking support in primary care. It consists of two components: (i) a tailored advice report generated by a computer program used by the smoking cessation advisor (SCA) during a primary care consultation; and (ii) a 3-month programme of automated tailored text messages sent to the participant’s mobile phone, designed to provide continuing support to participants during their quit attempt. A pilot trial with 602 participants from 32 practices (funded by NIHR School for Primary Care Research) found evidence for acceptability, feasibility and efficacy of the intervention compared with usual care alone (Naughton et al, 2014). Quit rate (self-reported prolonged abstinence at 6 months) was also significantly higher in the intervention group than in the usual care group (15.1% vs. 8.9%; p = .02; OR = 1.8; RR = 1.7), an effect that was if anything larger among the more socioeconomically deprived participants. Costing just £3.80 in text messages per smoker, if the above effect is confirmed, iQuit in Practice could prove to be a highly cost-effective intervention.

# Background

In the National Health Service (NHS), more smokers are treated in primary care than in other settings (227,624 in England in 2013-14). About 33% of these will be CO-validated quitters at 4 weeks, but only about 25% of these (8%) will still be abstinent at 12 months. Given the large number of smokers who are treated in primary care, even a small improvement in quit rate would produce a large increase in the number of successful long-term quitters. In order to respond to this issue, our research team developed and piloted iQuit in Practice as an intervention to be used alongside routine primary care smoking cessation support. The intervention builds on previous work by the research group (e.g. Sutton & Gilbert, 2007), and is designed to provide detailed and highly tailored advice to individual participants to help support their quit attempt.

*Tailored interventions*

Tailored interventions use data collected on or about an individual to make the information provided to them more personally relevant, increasing the likelihood that it will be read, understood, and acted upon. A Cochrane review of print based self-help interventions for smoking cessation (Hartmann-Boyce et al, 2014) identified 31 trials of tailored materials, which overall showed a small benefit (N = 40,890; RR 1.28, 95% CI 1.18 to 1.37). The evidence was strongest for tailored materials compared to no intervention, but also suggested that tailored materials are more effective than standard materials. The results from our own trial of tailored advice letters for smoking cessation were consistent with these findings in showing a small but useful effect of tailored advice letters on 6-month quit rates (Sutton & Gilbert, 2007).

*Text messaging*

An estimated 92% of UK adults own a mobile phone, and some 145 billion text messages were sent in the UK in 2013, averaging 170 per person per month (Ofcom report, 2014). Younger adults and people from lower socioeconomic groups are more likely to have a mobile phone as their sole form of telecommunication Ofcom, 2014). As such, text messaging is an important communication medium, and a potentially valuable tool for delivering smoking cessation advice and support to people across age and socioeconomic groups. While a number of SMS-based smoking cessation programmes have been found to be feasible and acceptable (Jamison et al, 2011) data on long-term effectiveness are limited (Whittaker et al, 2009). The UK txt2stop intervention was one study that did find a long-term effect of messages, with those receiving standard non-tailored messages more likely to be abstinent at six months than those in a no intervention control arm (Free et al, 2011). While txt2stop delivered standard messages, iQuit delivers text messages that are highly tailored to the individual, using both information collected at baseline, and subsequent data obtained from the smoker via two-way texting (dynamic tailoring).

*Use in primary care*

GP practices have access to a large number of patient records where patient smoking status is recorded, and nurses and GPs are encouraged to offer appropriate cessation advice (West, McNeill & Raw, 2000). In our earlier trial, smoking cessation advisors (SCAs) who used the iQuit program were largely positive about the intervention, reporting that it helped patients to engage with the consultation, and that it complemented current practice. Having already seen positive results in the pilot study, this pragmatic randomised control trial will allow us to decisively establish the effectiveness and cost-effectiveness of iQuit in Practice as an adjunct to usual care.

# The iQuit in Practice definitive trial

## Study objective:

The aim of this study is to assess the effectiveness and cost-effectiveness of the iQuit in Practice intervention compared with usual care alone in a definitive pragmatic trial with 6-month follow-up and biochemical verification of abstinence.

## Study design:

The study is a two parallel-group randomised controlled trial with 1:1 individual allocation comparing usual care (control) with usual care plus the iQuit system (intervention). Randomisation is stratified by SCA, so that each SCA will see approximately equal numbers of intervention and control participants. The allocation sequence is generated by a computer-based random number generator using random permuted blocks with block sizes of four and six to make the sequence difficult to predict, whilst avoiding a major imbalance between intervention and control groups if a block is incomplete at the end of recruitment. The sequence is stored on the web server database, accessible only to the data manager and the chief investigator. A cluster randomised design was considered for the trial, but decided against, as the randomisation built into the iQuit computer program is easily implemented, maintains allocation concealment and avoids selection bias. In addition, if there is clustering of outcomes, an individually randomised design requires a smaller sample size to detect an effect. Individual allocation worked effectively in the pilot trial, with no contamination found. Allocation is made by the web server during the consultation once the first part of the iQuit questionnaire is submitted (see procedure). At this point, the SCA and the participant become un-blinded to allocation. The nature of the trial makes blinding of the SCA or the participant unfeasible.

## Trial intervention

Control

‘Usual care’ will consist of routine smoking cessation advice delivered by SCAs. This includes a brief discussion about smoking habits and history, measurement of exhaled air carbon monoxide (CO), brief advice to quit, setting a quit date within the next 14 days, options for pharmacotherapy, and arranging a follow-up visit or contact to establish 4-week outcome. Usually the opportunity for multiple follow-up visits is offered.

Intervention

The intervention will consist of usual care, as described above, plus a tailored advice report and a programme of tailored text messages generated by the iQuit system.

*Advice report.*

The iQuit program utilises participant answers to the iQuit questionnaire to instantly produce a highly tailored detailed advice report, approximately three A4 pages in length. The system incorporates theories of smoking cessation and behaviour change, findings from previous studies, feedback from the earlier iQuit in Practice trial, and best practice guidance from the National Centre for Smoking Cessation Training. To ensure adequate tailoring, iQuit asks detailed questions about an individual’s smoking habits, including nicotine dependence, reasons for quitting, self-image, difficult situations, living with other smokers, social support and current health problems. During the consultation, the SCA asks the participant the questions and enters their answers into the iQuit computer program.

*Text messaging.*

A 90-day program of automatically generated text messages is sent to the participant’s mobile phone, beginning the day before their quit-date. Participants receive zero, one or two messages each day, with fewer messages towards the end of the 90-days. The messages are a further refinement of those developed for the iQuit pilot trial, and are designed to remind participants about their quit attempt, provide information about reasons for quitting, increase and maintain motivation, boost confidence at quitting, and provide coping strategies for difficult situations. Messages are individually tailored using baseline information and additional information obtained via query messages sent to participants throughout the 90 days. These messages ask if the participant has smoked in the last week, and participants can text ‘Y’ or ‘Yes’, ‘N’ or ‘No’. Participants can also at any time text ‘HELP’ if they are tempted to smoke, or ‘SLIP’ if they have had a lapse, to receive an immediate tailored support messageo stop receiving further messages, participants can text ‘STOP’ at any time. Participants can also email or telephone the study team to stop the messages if they would prefer. In our experience, smokers find it helpful to distract themselves occasionally from thinking about smoking and quitting smoking. The text messaging system allows the user to text ‘QUIZ’ to receive a general knowledge quiz question at any time. After submitting their answer, they receive a text telling them whether their answer is correct or incorrect. The quiz questions and answers are shown in Appendix 1.

## Sample size and power calculations

A total of 1452 participants will be recruited for this study. In the iQuit in Practice pilot trial, the quit rates (self-reported prolonged abstinence at 6 months) were 8.9% and 15.1% respectively (Naughton et al, 2014). If we assume a 90% response rate to biochemical verification and that 90% of these are confirmed as non-smokers (figure from StopAdvisor trial supplied by Dr Jamie Brown, UCL), this gives estimated validated quit rates of 7.1% and 12.1% for control and intervention respectively. An absolute increase of 5% quitting would be a worthwhile and scalable effect for this low cost intervention. Detecting an effect of this size with 90% power using a two-sided chi-squared test at the 5% significance level requires 726 participants per arm (non-responders at follow-up assumed to be smoking; Jackson et al, 2014),1452 in total.

Regular review of rates of follow-up observed the uptake of biochemical validation to be lower than previously estimated. It was calculated therefore that the trial analyses would retain 86% power even when allowing for biochemical validation uptake to be at a lower level of 70%, with an increased sample size of 1700, agreed by the Trial Steering Committee (TSC).

Therefore a total of 1700 participants will be recruited for this study (850 in each arm).

## Recruitment

### Practice recruitment

To be eligible for the trial, practices must have at least one member of staff (e.g. nurse, healthcare assistant) who provides stop smoking support as a smoking cessation advisor (SCA). The SCA(s) must have access to a computer, printer and internet in their consultation room. The practice should not be participating in any other smoking cessation research study, and preference is given to those geographically accessible from Cambridge. Based on the required sample, we anticipate recruiting 66 practices for the study, recruiting 22 participants each. This is based on the iQuit in Practice pilot trial, in which practices recruited on average 2.5 participants each per month over the recruiting period. Practices will represent a range of demographic and deprivation scores.

Training provided for practice staff

The SCAs will receive training on the iQuit computer program, the research intervention, and on taking informed consent. Training will also cover managing confidentiality within a research setting, and collecting and recording accurate, explicit and complete data for participants. Training will take place in a single 2-hour session in the practice. Follow-up iQuit training and ongoing support is available on a flexible basis, based on individual practice SCAs’ needs.

### Participant recruitment

Inclusion Criteria

A patient can be included in the study if they meet the following inclusion criteria:

- Current smoker (usually smokes at least one cigarette a day and has smoked in the 7 days prior to randomisation date)
- Able to speak English and can provide written informed consent
- Wants to quit smoking and is willing to set a quit date within the 14 days after randomisation
- Aged 18 or over
- Has a mobile phone and is familiar with sending and receiving SMS text messages
- Is willing to participate in the iQuit study and follow study procedures
- Is not currently involved in another formal smoking cessation study or program

Exclusion Criteria

Patients are excluded if they do not meet all the inclusion criteria. Patients will also be excluded if considered by their GP to be unsuitable for other reasons such as severe mental impairment or severe or terminal illness. Patients with co-morbidities, for example chronic obstructive pulmonary disease (COPD), or diabetes, are not excluded from the study unless their GP considers them unsuitable. In addition, a person living in the same household as an existing trial participant will not be excluded from participating in the trial, assuming that they meet the inclusion/exclusion criteria.

### Recruitment procedure

Participants are recruited via two means:

1. Opportunistic recruitment: patients who self-refer, or who are referred via another health professional to the SCA for smoking cessation advice, are given (or sent via post) a Participant Information Sheet (PIS) by a member of practice reception staff. Self-referral will be encouraged through the use of posters and leaflets on display in the practice waiting area.
2. Proactive recruitment: potential participants are identified via smoking status data collected by GP surgeries during routine medical appointments, and a list of potential participants is generated from the practice database by a member of practice staff. A random selection of those potentially eligible to participate is sent a covering letter from the patient’s GP, and a PIS. Potentially eligible patients might also be sent an SMS text message informing them about the study and advising them to contact the surgery for further details.

In both instances, those interested in participating in the research are asked to make an appointment with the relevant SCA at the GP practice. Potential participants who fail to attend their initial appointment with the SCA are sent a reminder letter via the GP practice, prompting them to make another appointment. If the potential participant does not respond, or does not attend a second appointment, there is no further follow-up by the practice or the research team.

## Informed Consent

With the procedure outlined above, potential participants should have received the participant information sheet (PIS) at least 24 hours prior to their appointment. At the first appointment, the SCA will confirm that the potential participant received a copy of the PIS, and if so, will check eligibility and answer any questions. In the event that a potential participant did not receive the PIS, they will have the study fully explained to them by the SCA, and be invited to take part. This is in order to ensure equal opportunity to participate, as it would be unethical not to offer the opportunity to those potentially interested due to not receiving the PIS beforehand. Those who are eligible and who wish to participate are asked to sign a participant consent form. The original signed form is kept by the research team, a copy given to the participant, and a copy kept on the participant medical record at the practice. Those who are ineligible or who do not wish to participate will receive usual care and follow-up.

Consort reporting requirements

In line with the reporting requirements of the CONSORT (Consolidated Standards of Reporting Trials) Statement (Altman et al., 2001; Moher at al., 2001), we will collect data on the number of participants approached to take part in the trial, the number who are eligible, and the reasons for non-participation. Information about eligibility collected using an online system. When the SCA sees a new patient who is interested in taking part, he/she opens the online CRF and checks the patient’s eligibility. If they are ineligible, the reason is recorded. If they are eligible, written informed consent is obtained, and the SCA asks the participant to complete a brief health questionnaire (EQ-5D).

## Consultation with the Smoking Cessation Advisor following consent to participate

The SCA provides ‘usual care’ to all consenting participants, including advice on smoking cessation, measuring carbon monoxide levels, exploring pharmacotherapy, and supporting the participant to set a quit-date in the next two weeks. Then, in consultation with the participant, the SCA enters answers to the questions on part one of the iQuit Questionnaire which is part of the online CRF. These include cigarettes a day and motivation to quit. The SCA will also input the participant’s Carbon monoxide (CO) level. The CO level is used to characterise the sample and check baseline comparability between the two groups. It will also be used in the tailored advice report for participants receiving the intervention.

Once part 1 of the iQuit questionnaire is completed, the iQuit program will randomise the participant to either the control group or the intervention group. For participants randomised to the control group, no further action is required, and the SCA will conclude the consultation. For those in the intervention group, the SCA will go through part 2 of the iQuit questionnaire with the participant and the iQuit program will use the responses to these questions to automatically generate the tailored advice report, which the SCA will then print and give to the participant. Participants in the intervention group will also begin to receive text messages commencing one day before their quit date. All further consultations from this date will continue routinely for participants in both groups.

## Frequency and duration of participant follow-up

Every participant will be contacted initially by text or email at 6 months from randomisation, with a brief question asking about their current smoking status (primary outcome question). Following a response, participants will be sent a link to complete the remaining questions on-line or given the opportunity to receive the questionnaire by post. If there is no response by these means we shall endeavour to follow up participants through a telephone call. Should a participant not respond after six telephone contact attempts, and they have not responded to the primary outcome question by text, email, or online or postal version of the survey, they will be recorded as lost to follow-up. For the main analysis, participants who cannot be contacted at follow-up will be assumed to be smoking (intention-to-treat analysis).

**5.8.1 Saliva samples**

All those who state that they are abstinent from smoking and have smoked no more than 5 cigarettes in total during the follow-up period are asked to provide a saliva sample. The sample is collected by placing a swab underneath the tongue. Participants are sent a kit containing full instructions for collecting a sample and a padded freepost envelope for sending it to the laboratory (ABS Laboratories, Welwyn Garden City, Hertfordshire) for analysis. The laboratory will analyse the sample for cotinine, a metabolite of nicotine, and, if the participant reports having used NRT or e-cigarettes in the last 7 days, anabasine (to differentiate whether the cotinine has come from tobacco, E-Cig or NRT). Once analysed, the sample will be destroyed in accordance with “the Human Tissue Act (HTA). The study team are not directly involved in the handling of the saliva sample. A £5 voucher is included in the letter to the participant to compensate them for the inconvenience. The letter also includes a short questionnaire asking the participant if they have smoked tobacco, used e-cigarettes or used a nicotine replacement product in the last 7 days; the participant is asked to return this with their sample.

In cases where a saliva sample has not been received by the laboratory after 10 days, participants will be phoned by a member of the research team and asked to provide a sample. Where no saliva sample is returned or the participant is unwilling to provide a sample, they will be encouraged to make an appointment with their GP surgery to undergo a carbon monoxide breath test with their SCA, as an alternative to a saliva sample. The CO breath test is part of a routine smoking cessation consultation, takes only about 5 minutes to do and is familiar to both patients and SCAs. However, a participant is free to refuse either form of biomedical validation.

**5.8.2 Ethnicity, occupation and 4-week quit outcome**

At the end of the study, ethnicity, occupation details and the 4-week quit outcome will be collected from GP practices for all participants.

## Confidentiality

Information obtained throughout the study will remain strictly confidential. All data is stored securely in accordance with the Data Protection Act and the University of Cambridge policy on data security. Electronic data is stored on a secure partition on a University of Cambridge file server. Only the research team will have access to data, with the following exceptions:

1. The text messages are transferred by a company called FastSMS. For participants in the intervention group, the company will have access to name (the first name is used in some of the messages), mobile phone number and the content of the message. The company privacy policy states that all information will remain confidential and will not be disclosed to any third parties.
2. A data processing company might be used to process anonymised data from questionnaire responses. The questionnaires will not contain any identifiable data.
3. ABS laboratories; however the saliva sample they receive will include only the study ID for identification purposes.
4. It is possible that auditors from the funder, sponsor, NHS Trust and regulatory inspectors may require access to the data to check that that the study is being properly conducted.
5. Requests for datasets may be received from outside researchers. If such requests are received at least 3 years after the study has finished (April 2023), they will be considered by the project data committee (CI, trial manager and data manager) and only data with all identifiable personal data removed will be released.

After the study has ended, consent forms and anonymised data may be stored for a minimum of 10 years, to ensure that it has been properly reported. Secure storage will be in accordance with national guidance. Data will be removed and destroyed if participants choose to withdraw from the study and do not wish for their anonymised data to be used.

## Measures

The primary outcome measure is self-reported prolonged abstinence over the whole 6-month follow-up period (allowing for up to five cigarettes in total), combined with 7-day point prevalence with biochemical verification at 6 months to conform to the Russell Standard (West et al, 2005).

Secondary outcome measures include:

1. CO-verified abstinence at 4-week quit date follow-up for at least 2 weeks, assessed by the SCA.
2. Self-reported prolonged abstinence over the whole of the 6-month follow-up period (allowing for up to five cigarettes in total)
3. Self-reported 7 day point-prevalence abstinence at 6-month follow-up.
4. Cost and utility measures: time required to complete iQuit questionnaire; resource use (including use of cessation medication); EQ-5D.

Process measures (all recorded at 6 months) include:

1. number of serious quit attempts lasting at least 24 hours during the 6-month follow-up period.
2. motivation and confidence in quitting.
3. Use of strategies to help avoid smoking or lapsing during the 6-month follow-up period.
4. (intervention group only) evaluation of the tailored advice report and text messaging programme (e.g. how helpful they found them; how they felt about the number of texts sent etc.).

## Statistical analysis

The two groups will be compared using χ2 tests and logistic regression analysis, adjusting for stratifiers and any imbalances in potential confounders between groups. The results will be reported in terms of the absolute difference in percentages or proportions and the odds ratio, with 95% confidence intervals. Analysis will be intention-to-treat, where all those randomised are analysed and participants lost to follow-up assumed to be smoking. We will also conduct sensitivity analyses using a range of less severe assumptions, including a complete-case analysis (Jackson et al, 2014). Deprivation (based on IMD derived from participant’s home postcode) will be examined as a potential moderator of the intervention effect by including a multiplicative term in the logistic regression analysis.

## Time and event schedule for participants

| Time / Event | Randomisation  (day 0) | Quit date set by patient* (Day 1 to 14) | Intervening days | 4 weeks after quit date (routine NHS follow-up) | Intervening days | 6 months after randomisation date |
| --- | --- | --- | --- | --- | --- | --- |
| Attend appointment with SCA, at GP practice | X |  |  | X |  |  |
| Written consent obtained to participate in ‘iQuit in Practice’ research study | X |  |  |  |  |  |
| SCA provides ‘usual care’ including CO monitoring | X |  |  | X |  |  |
| Complete on-line iQuit questionnaire with SCA | X |  |  |  |  |  |
| Randomisation to either control or intervention group | X |  |  |  |  |  |
| Receive tailored advice print-out (intervention group only) | X |  |  |  |  |  |
| Participant starts quit attempt |  | X |  |  |  |  |
| Receive tailored text-messages (intervention group only). |  | X  (starting 1 day before quit date) | X | X | X (till 90 days after quit date) |  |
| SCA completes routine 4-week follow-up, as per NHS guidance |  |  |  | X |  |  |
| Follow-up text/email/ questionnaire/telephone call from co-ordinating centre in Cambridge. |  |  |  |  |  | X |
| Saliva sample kit sent to all participants who state prolonged abstinence |  |  |  |  |  | X |

SCA = Smoking Cessation Advisor

* The quit date must be set within the 14 days after randomisation.

Events highlighted in grey are only received by participants randomised to the ‘intervention’ group.

# Trial Management and Governance Arrangements

## Research Team

| Chief Investigator: | Professor Stephen Sutton | University of Cambridge |
| --- | --- | --- |
| Research team: | Felix Naughton | University of East Anglia |
|  | Jo Mitchell | University of Cambridge |
|  | Melanie Sloan | University of Cambridge |
|  | Sarah Hopewell | University of Cambridge |
|  | Miranda van Emmenis | University of Cambridge |
|  | James Brimicombe | University of Cambridge |
| Principal Investigators: | Smoking Cessation Advisors | GP practices |

The day-to-day management of the trial is performed by the Research Team based at the Institute of Public Health, University of Cambridge, Forvie Site, CB2 0SR. The team will hold fortnightly trial management meetings in order to discuss ongoing progress and any amendments required. Any correspondence should be addressed to the Chief Investigator.

## Sponsor

The ‘Sponsor’ is defined as the “Individual, organisation or group taking on responsibility for securing the arrangements to initiate, manage and finance a study”. The University of Cambridge will act as Sponsor for the trial.

## Study Agreements

Written agreements detailing responsibilities for the trial are signed by parties prior to participant recruitment. These will include the responsibilities of the sponsor, the research team, and the principal investigators, and are initiated by the Research Services Division of the University of Cambridge.

## Study Funding and NHS Cost implications

The research costs for the trial are funded through a 4-year project grant awarded by Cancer Research UK. NHS Service Support Costs include the additional NHS patient-related costs associated with the study. For ‘iQuit in Practice’, this includes the extra time required of NHS staff in order to: attend ‘start-up’ meetings; attend training to undertake the research; identify patients’ eligible to participate; send letters out to patients; explain the study to participants; obtain informed consent; and collect research-specific data.

## Adoption of ‘iQuit in Practice’ onto the UKCRN Clinical Research Portfolio

The iQuit trial has been adopted by the UKCRN and additional NHS Service Support has been sought and approved by the East of England Primary Care Research Network, The study team uploads information on the number of participants recruited to the NIHR CRN accruals database.

## Insurance/Indemnity

The University of Cambridge insurance office has been consulted to ensure that appropriate insurance/indemnity arrangements are in place to meet the potential legal liability of the Sponsor, and investigators/collaborators arising from harm to participants in the design, management and conduct of the research. Since participants are NHS patients, indemnity is provided through NHS schemes or through professional indemnity. GP Practices must check with their providers of insurance that participation in the research is covered for negligent harm.

## Adverse Incident reporting

Since iQuit in Practice does not fall under the Medicines for Human Use (Clinical Trials) Regulations 2004 (as amended), the research team will not collect data on adverse events (AEs), serious adverse events (SAEs) or suspected unexpected serious adverse reactions (SUSARs) as defined by these regulations. However, adverse incidents could still happen, for example;

- breach of confidentiality
- patient complains about aspect of treatment as a study participant
- deviation from study protocol (e.g. recruiting before consent)
- equipment failure
- aggressive behaviour from a participant towards the researcher, practice staff or others.

Adverse incidents relating to the conduct of this research must be reported to the study team within 5 working days of the researcher becoming aware of the incident, using an ‘adverse incident report form’. Incidents of a serious nature may need to be reported to the appropriate study REC.

## Intellectual Property (IP)

The web-based iQuit software has been developed over several years by Professor Stephen Sutton (University of Cambridge), Dr Hazel Gilbert (University College London) and colleagues. The software is protected by a copyright statement. Further IP advice, regarding protection and commercialisation, will be sought from Cambridge Enterprise (the technology transfer company for the University of Cambridge) as required.

## Trial Steering Committee.

A Trial Steering Committee (TSC), has been convened and will meet on a 6-12 monthly basis as necessary to oversee the running of the study in terms of quality, budget, timescales etc. Terms of Reference have been developed and adopted by the Committee to outline its role and responsibilities. The trial steering committee comprises the following members:

Independent Chair: Jamie Brown (UCL)

Independent statistician: Tom Fanshawe (University of Oxford)

Independent member: Irwin Nazareth (UCL)

Sponsor representative: Carolyn Read

PPI representative: Dan Tarrant-Willis

CI

Trial manager

## Research & Development and Research Ethics Committee (REC) approvals

Written approval has been obtained from the Eastern CRN, Cambridge East REC (Ref no: 188824) and the HRA. Any subsequent amendments to study documentation, along with safety, annual progress and final study reports will be submitted to the HRA and REC for information and/or approval, as required. All data will be processed in accordance with the Data Protection Act 1998.

## Patient and Public Involvement

Small focus groups of smokers and ex-smokers assisted with the development of the original iQuit in Practice text message program, and PPI representatives were invited to comment on any revisions prior to study commencement. PPIs will also be consulted on dissemination of the study findings.

## Dissemination of research results

Information on the progress of the iQuit in Practice trial will be publicly available via a number of organisation websites including:

| University of Cambridge PCU site: | http://www.phpc.cam.ac.uk/pcu/ |
| --- | --- |
| UKCRN Portfolio: | http://public.ukcrn.org.uk/search/ |
|  |  |
| ISRCTN Register: | http://isrctn.org/ |

Results of the research will be published in peer-reviewed journals and findings presented to a wide audience, including national and international conferences and other scientific meetings. The 6-month follow-up form will include the opportunity for participants to request a report of the results. A lay summary will be prepared and sent to all participants requesting one.

## Proposed study timetable

|  | Year 1 | | | | Year 2 | | | | Year 3 | | | | Year 4  Year 5 | | | |  | |
| --- | --- | --- | --- | --- | --- | --- | --- | --- | --- | --- | --- | --- | --- | --- | --- | --- | --- | --- |
|  | Jan-Mar 2016 | April-June 2016 | July-Sept 2016 | Oct-Dec 2016 | Jan-Mar 2017 | April-June 2017 | July-Sept 2017 | Oct-Dec 2017 | Jan-Mar 2018 | April-June 2018 | July-Sept 2018 | Oct-Dec 2018 | Jan-Mar 2019 | April-June 2019 | July-Sept 2019 | Oct-Dec 2019 | Jan-Mar 2020 | April-June 2020 |
| Recruit practices |  |  |  |  |  |  |  |  |  |  |  |  |  |  |  |  |  |  |
| Train practice staff to deliver interventions |  |  |  |  |  |  |  |  |  |  |  |  |  |  |  |  |  |  |
| Participant recruitment period |  |  |  |  |  |  |  |  |  |  |  |  |  |  |  |  |  |  |
| Delivery of intervention |  |  |  |  |  |  |  |  |  |  |  |  |  |  |  |  |  |  |
| 6-month follow-up |  |  |  |  |  |  |  |  |  |  |  |  |  |  |  |  |  |  |
| Biochemical verification |  |  |  |  |  |  |  |  |  |  |  |  |  |  |  |  |  |  |
| Data analysis/write-up & dissemination. |  |  |  |  |  |  |  |  |  |  |  |  |  |  |  |  |  |  |

References

Altman DG, Schulz KF, Moher D, Egger M. Davidoff F, Elbourne D, Gøtzsche PC, Lang T. The revised CONSORT statement for reporting randomized trials: explanation and elaboration. Ann Intern Med 2001; 134(8):663-694.

Free C, Knight R, Robertson S, Whittaker R, Edwards P, Zhou W, Rodgers A, Cairns J, Kenward MG, Roberts I. Smoking cessation support delivered via mobile phone text messaging (txt2stop): a single‐blind, randomised trial. *Lancet* 2011;378:49–55.

Gilbert H, Nazareth I, Sutton S. Assessing the feasibility of proactive recruitment of smokers to an intervention in general practice for smoking cessation using computer-tailored feedback reports. Family Practice, 2007, 24, 388-394.

Gilbert H, Nazareth I, Sutton S, Morris R, Godfrey. Effectiveness of computer-tailored smoking cessation advice in primary care (ESCAPE): A randomised trial. Trials, 2008, 9, 23.

Hartmann-Boyce J, Lancaster T, Stead LF. Print-based self‐help interventions for smoking cessation. Cochrane Database Syst Rev 2014, Issue 6. Art. No.: CD001118. DOI: 10.1002/14651858.CD001118.pub3.

Jackson D, White IR, Mason D, Sutton S. A general method for handling missing binary outcome data in randomised controlled trials. *Addiction,* published online Aug 29 2014.

Jamison J, Sutton S, Gilbert H: Delivering tailored smoking cessation support via mobile phone text messaging: A feasibility and acceptability evaluation of the Quittext program. *J Appl Biobehav Res* 2012;17:38–58.

Moher D, Schulz KF, Altman DG. The CONSORT statement: revised recommendations for improving the quality of reports of parallel-group randomised trials. Lancet 2001; 357(9263):1191-1194.

Naughton F, Jamison J, Boase S, Sloan, Gilbert H, Prevost AT, Mason D, Smith S, Brimicombe J, Evans R, Sutton S. Randomized controlled trial to assess the short-term effectiveness of tailored web- and text-based facilitation of smoking cessation in primary care (iQuit in Practice). Addiction 2014 109:1184-93.

Ofcom. Communications Market Report. 2013. <http://stakeholders.ofcom.org.uk/binaries/research/cmr/cmr13/2013_UK_CMR.pdf>

Stead LF, Lancaster T. Group behaviour therapy programmes for smoking cessation. Cochrane Database of Systematic Reviews 2005, Issue 2. Art. No.: CD001007. DOI: 10.1002/14651858.CD001007.pub2.

Sutton S, Gilbert H. Effectiveness of individually-tailored smoking cessation advice letters as an adjunct to telephone counselling and generic self-help materials: randomized controlled trial. Addiction, 2007, 107, 994-1000.

West R, McNeill A, Raw M: Smoking cessation guidelines for health professionals: an update. Thorax 2000, 55:987-99.

Whittaker R, Borland R, Bullen C, Lin RB, McRobbie H, Rodgers A: Mobile phone‐based interventions for smoking cessation. Cochrane Database Syst Rev 2009, 4:CD006611. doi:10.1002/14651858.CD006611.pub2.

# Appendix 1: QUIZ questions

**Quiz question**

QUIZ: How many eyes do starfish have? Reply A1 for none, A2 for 4 or A3 for the same number as they have arms.

Response

Incorrect! Starfish have one eye per arm, meaning they vary from 5 to 50 eyes! The eyes aren't anywhere near as good as human eyes, but can see basic images.

Incorrect! Starfish have one eye per arm, meaning they vary from 5 to 50 eyes! The eyes aren't anywhere near as good as human eyes, but can see basic images.

Correct! Starfish have one eye per arm, meaning they vary from 5 to 50 eyes! The eyes aren't anywhere near as good as human eyes, but can see basic images.

no response

Starfish have one eye per arm, meaning they vary from 5 to 50 eyes! The eyes aren't anywhere near as good as human eyes, but can see basic images.

**Quiz question**

How long did it take Neil Armstrong to get to the moon? A1 = 2 days 4 hours and 18 mins, A2 = 4 days 5 hours and 45 mins, A3 = 7 days 16 hours and 12 mins.

Response

Incorrect! It took 4 days 6 hours and 45 mins for Apollo 11 to take Neil Armstrong, Michael Collins and Buzz Aldrin to the moon. They stayed for about 22 hours.

Correct! It took 4 days 6 hours and 45 mins for Apollo 11 to take Neil Armstrong, Michael Collins and Buzz Aldrin to the moon. They stayed for about 22 hours.

Incorrect! It took 4 days 6 hours and 45 mins for Apollo 11 to take Neil Armstrong, Michael Collins and Buzz Aldrin to the moon. They stayed for about 22 hours.

No response

It took 4 days 6 hours and 45 mins for Apollo 11 to take Neil Armstrong, Michael Collins and Buzz Aldrin to the moon. They stayed for about 22 hours.

**Quiz question**

QUIZ: When was Facebook launched? Reply A1 for December 2001, A2 for February 2004, or A3 for June 2008.

Response

Incorrect! Facebook was launched on 4th February 2004. At first it was just for university students, but since 2006, anyone over 13 can register.

Correct! Facebook was launched on 4th February 2004. At first it was just for university students, but since 2006, anyone over 13 can register.

Incorrect! Facebook was launched on 4th February 2004. At first it was just for university students, but since 2006, anyone over 13 can register.

No response

Facebook was launched on 4th February 2004. At first it was just for university students, but since 2006, anyone over 13 can register.

**Quiz question**

QUIZ: How old was the oldest man to run the London Marathon? Reply A1 for 84, A2 for 89, or A3 for 93

Response

Incorrect! Fauja Singh ran it aged 93 in 2004, completing it in 6 hours 7 minutes. He since ran the Toronto Marathon in 8 hours 11 mins in 2011, aged 100.

Incorrect! Fauja Singh ran it aged 93 in 2004, completing it in 6 hours 7 minutes. He since ran the Toronto Marathon in 8 hours 11 mins in 2011, aged 100.

Correct! Fauja Singh ran it aged 93 in 2004, completing it in 6 hours 7 minutes. He since ran the Toronto Marathon in 8 hours 11 mins in 2011, aged 100.

no response

Fauja Singh ran it aged 93 in 2004, completing it in 6 hours 7 minutes. He since ran the Toronto Marathon in 8 hours 11 mins in 2011, aged 100.

**Quiz question**

QUIZ: When was the first ever text message sent? Reply A1 for 1987, A2 for 1992, or A3 for 1995

Response

Incorrect! The first text message was sent on 3rd December 1992 by Neil Papworth. He typed it on a computer and sent it to a colleague at a Christmas party.

Correct! The first text message was sent on 3rd December 1992 by Neil Papworth. He typed it on a computer and sent it to a colleague at a Christmas party.

Incorrect! The first text message was sent on 3rd December 1992 by Neil Papworth. He typed it on a computer and sent it to a colleague at a Christmas party.

no response

The first text message was sent on 3rd December 1992 by Neil Papworth. He typed it on a computer and sent it to a colleague at a Christmas party.

**Quiz question**

QUIZ: What is the recorded highest number of children parented by one couple? Reply A1 for 24, A2 for 33, or A3 for 69.

**Response**

Incorrect! Mr and Mrs Vassilyev are recorded to have had 69 children between 1725 and 1765, including twins, triplets and quadruplets. 67 survived infancy.

Incorrect! Mr and Mrs Vassilyev are recorded to have had 69 children between 1725 and 1765, including twins, triplets and quadruplets. 67 survived infancy.

Correct! Mr and Mrs Vassilyev are recorded to have had 69 children between 1725 and 1765, including twins, triplets and quadruplets. 67 survived infancy.

**no response**

Mr and Mrs Vassilyev are recorded to have had 69 children between 1725 and 1765, including twins, triplets and quadruplets. 67 survived infancy.

**Quiz question**

**QUIZ:** Which act has had the most number 1 UK singles? Reply A1 for Elvis Presley, A2 for The Beatles, or A3 for Madonna

**Response**

Correct! Elvis Presley currently holds the top spot, having had 21 UK number 1 singles.

Incorrect! Elvis Presley currently holds the top spot, having had 21 UK number 1 singles.

Incorrect! Elvis Presley currently holds the top spot, having had 21 UK number 1 singles.

**no response**

Elvis Presley currently holds the top spot, having had 21 UK number 1 singles.

**Quiz question**

**QUIZ**: How much is Kate Middleton's engagement ring believed to be worth? Reply A1 for £100,000, A2 =£300,000, A3 = £500,000

**Response**

Incorrect! It is worth around £300,000. The ring originally belonged to Princess Diana and has an 18 carat sapphire and 14 diamonds

Correct. The ring originally belonged to Princess Diana and has an 18 carat sapphire and 14 diamonds

Incorrect! It is worth around £300,000. The ring originally belonged to Princess Diana and has an 18 carat sapphire and 14 diamonds

**no response**

Kate Middleton's engagement ring is worth around £300,000. The ring originally belonged to Princess Diana and has an 18 carat sapphire and 14 diamonds

**Quiz question**

**QUIZ:** When was the first stretch of motorway built in the UK? Reply A1 for 1958, A2 for 1965, or A3 for 1973

**Response**

Correct! The first UK motorway was the Preston Bypass, now part of the M6, and opened on 5th December 1958. It was 8 miles long and had no hard shoulder.

Incorrect! The first UK motorway was the Preston Bypass, now part of the M6, and opened on 5th December 1958. It was 8 miles long and had no hard shoulder

Incorrect! The first UK motorway was the Preston Bypass, now part of the M6, and opened on 5th December 1958. It was 8 miles long and had no hard shoulder

**no response**

The first UK motorway was the Preston Bypass, now part of the M6, and opened on 5th December 1958. It was 8 miles long and had no hard shoulder

**Quiz question**

QUIZ: Which is the heaviest land mammal? Reply A1 for African elephant, A2 for white rhinoceros or A3 for hippopotamus.

**Response**

Correct! The African elephant is the heaviest land mammal, weighing up to 6 tons. That's about three cars! They eat up to 450kg per day of vegetation.

Incorrect! The African elephant is the heaviest land mammal, weighing up to 6 tons. That's about three cars! They eat up to 450kg per day of vegetation.

Incorrect! The African elephant is the heaviest land mammal, weighing up to 6 tons. That's about three cars! They eat up to 450kg per day of vegetation.

**no response**

The African elephant is the heaviest land mammal, weighing up to 6 tons. That's about three cars! They eat up to 450kg per day of vegetation.

**Quiz question**

**QUIZ:** What is the deepest sea in the world? Reply A1 for the Irish Sea, A2 = the Red Sea, A3 =the Caribbean

**Response**

Incorrect! It's the Caribbean. The world's deepest point is the Mariana Trench. If you dropped Mount Everest into it, its peak would still be a mile underwater!

Incorrect! It's the Caribbean. The world's deepest point is the Mariana Trench. If you dropped Mount Everest into it, its peak would still be a mile underwater!

Correct. The world's deepest point is the Mariana Trench. If you dropped Mount Everest into it, its peak would still be a mile underwater!

**no response**

It's the Caribbean. The world's deepest point is the Mariana Trench. If you dropped Mount Everest into it, its peak would still be a mile underwater!

**Quiz question**

**QUIZ:** What's the average pay for a UK premier league footballer? Reply A1 for £20,000 per week, A2 for 40,000 per week, or A3 for £60,000 per week.

**Response**

Incorrect! The average premier league player earns about £40,000 per week. However, some players earn much more. Wayne Rooney earned £260,000 per week in 2015.

Correct! The average premier league player earns about £40,000 per week. However, some players do earn much more. Wayne Rooney earned £260,000 per week in 2015.

Incorrect! The average premier league player earns about £40,000 per week. However, some players earn much more. Wayne Rooney earned £260,000 per week in 2015.

**no response**

The average premier league player earns about £40,000 per week. However, some players earn much more. Wayne Rooney earned £260,000 per week in 2015.

**Quiz question**

**QUIZ:** Which food item can, in the right conditions, be turned into diamonds? Reply A1 for coffee, A2 for ginger, or A3 for peanut butter

**Response**

Incorrect! Scientists have recently been using temperature and pressure to turn peanut butter into diamonds. Sadly it's complicated and can't be done at home!

Incorrect! Scientists have recently been using temperature and pressure to turn peanut butter into diamonds. Sadly it's complicated and can't be done at home!

Correct! Scientists have recently been using temperature and pressure to turn peanut butter into diamonds. Sadly it's complicated and can't be done at home!

**no response**

Scientists have recently been using temperature and pressure to turn peanut butter into diamonds. Sadly it's complicated and can't be done at home!

**Quiz question**

**QUIZ:** How long is the Channel Tunnel? Reply A1 for 31 miles, A2 for 43 miles, or A3 for 49 miles.

**Response**

Correct! The Channel Tunnel, linking England and France, is 31.4 miles long, and has 23.5 miles under the sea.

Incorrect! The Channel Tunnel, linking England and France, is 31.4 miles long, and has 23.5 miles under the sea.

Incorrect! The Channel Tunnel, linking England and France, is 31.4 miles long, and has 23.5 miles under the sea.

**no response**

The Channel Tunnel, linking England and France, is 31.4 miles long, and has 23.5 miles under the sea.

**Quiz question**

**QUIZ:** What did people in Victorian Britain who couldn't afford a chimney sweep use to clean their chimney? Reply A1 = Geese, A2 = Cats, A3 = Foxes.

**Response**

Correct! A live goose's legs were tied together before it was pushed up the flue. The goose would flap its wings in panic, loosening the soot.

Incorrect! A live goose's legs were tied together before it was pushed up the flue. The goose would flap its wings in panic, loosening the soot.

Incorrect! A live goose's legs were tied together before it was pushed up the flue. The goose would flap its wings in panic, loosening the soot.

**no response**

A live goose's legs were tied together before it was pushed up the flue. The goose would flap its wings in panic, loosening the soot.

**Quiz question**

QUIZ: What sense does Ben Cohen (one half of the famous ice cream duo Ben and Jerry) not have? Reply A1 = Sight, A2 = Smell, A3 = Hearing.

**Response**

Incorrect! Ben has anosmia (no sense of smell), which means he cannot taste well and needs lots of texture in his food. That's why their ice cream is so chunky.

Correct! Ben has anosmia (no sense of smell), which means he cannot taste well and needs lots of texture in his food. That's why their ice cream is so chunky.

Incorrect! Ben has anosmia (no sense of smell), which means he cannot taste well and needs lots of texture in his food. That's why their ice cream is so chunky.

**no response**

Ben has anosmia (no sense of smell), which means he cannot taste well and needs lots of texture in his food. That's why their ice cream is so chunky.

**Quiz question**

QUIZ: How many bones are there in a giraffe's neck? Reply A1 for 7, A2 for 15, or A3 for 21.

**Response**

Correct! Like all other mammals, a giraffe has 7 bones in its neck. However, these bones are very long, with each measuring about 25cm!

Incorrect! Like all other mammals, a giraffe has 7 bones in its neck. However, these bones are very long, with each measuring about 25cm!

Incorrect! Like all other mammals, a giraffe has 7 bones in its neck. However, these bones are very long, with each measuring about 25cm!

**no response**

Like all other mammals, a giraffe has 7 bones in its neck. However, these bones are very long, with each measuring about 25cm!

**Quiz question**

**QUIZ:** Which is the UK's longest running TV soap opera? Reply A1 for Emmerdale, A2 for Eastenders, or A3 for Coronation Street

**Response**

Incorrect! Coronation Street is the longest, running since 9th December 1960. Emmerdale started 16th October 1972, and Eastenders began 19th February 1985.

Incorrect! Coronation Street is the longest, running since 9th December 1960. Emmerdale started 16th October 1972, and Eastenders began 19th February 1985.

Correct! Coronation Street began on 9th December 1960. Emmerdale started on 16th October 1972, and Eastenders began on 19th February 1985.

**no response**

Coronation Street began on 9th December 1960. Emmerdale started on 16th October 1972, and Eastenders began on 19th February 1985.

**Quiz question**

For which film was Leonardo DiCaprio awarded an Oscar for Best Actor in a Leading Role? Reply A1 =Wolf of Wall Street, A2 =Blood Diamond, or A3 =The Revenant.

**Response**

Incorrect! DiCaprio was nominated for all three films, but received the award for his role in The Revenant in 2016.

Incorrect! DiCaprio was nominated for all three films, but received the award for his role in The Revenant in 2016.

Correct! DiCaprio was nominated for all three films, but received the award for his role in The Revenant in 2016.

**no response**

DiCaprio was nominated for all three films, but received the award for his role in The Revenant in 2016.

**Quiz question**

**QUIZ:** Which is the most southerly place in the UK? Reply A1 for Lands End, A2 for Lizard Point, or A3 for the Isle of Wight

**Response**

Incorrect! Lizard Point is the most southerly place in the UK, while Lands End is the most westerly.

Correct! Lizard Point is the most southerly place in the UK, while Lands End is the most westerly.

Incorrect! Lizard Point is the most southerly place in the UK, while Lands End is the most westerly.

**no response**

Lizard Point is the most southerly place in the UK, while Lands End is the most westerly.
