# Supplementary material for: Assessment of the Effectiveness and Cost-Effectiveness of Tailored Web- and Text-Based Smoking Cessation Support in Primary Care (iQuit in Practice II): Protocol for a Randomized Controlled Trial
Source: JMIR Res Protoc. 2020 Jul 14;9(7):e17160. doi: 10.2196/17160 (PMC7388034; doi:10.2196/17160)
Supplement: Multimedia Appendix 8 [file resprot_v9i7e17160_app8.pdf]

Cancer Research UK
Peer Review Comments

External Reveiwer 1

This page will be fed back to the applicant intact; please do not include any reference to your identity, or any particularly sensitive comments.

Importance of the proposal and its suitability for funding by Cancer Research UK
Please comment on the importance of the proposed work, its likely impact in the field of cancer research, and its suitability for funding by Cancer Research UK.

Effective tobacco control is by far the most important strategy for reducing cancer in the short to medium term at least. Thus any project that has good prospects of reducing smoking should be of the utmost priority for cancer control funders. This is an extremely interesting and important proposal. It is a trial of automated program for smoking cessation by primary health care providers. If it shows the expected effects, and as I argue below there are very strong reasons for expecting a positive outcome, it has the potential to provide a quantum improvement in the routine treatment of smoking within primary health care settings.

Originality of the work
Please comment on the novelty of the proposed studies. Have they been duplicated by others in the field?

This is a highly innovative proposal. The innovation comes from taking an extremely promising automated program into a primary health care setting and adding it to the ordinary care that is currently delivered. If successful, the trial is likely to provide a highly cost-effective strategy for improving the effectiveness of primary health care based smoking cessation. One of the identified limitations of automated programs is the lack of engagement with them by many who start using. Use beginning in a setting where the SCA can provide a framework for action and the motivating potential of a health professional should result in high levels of engagement, which is likely to maximise the potential benefits of the program. It is thus likely to add to the base quitting that is provided by the use of pharmacotherapy and the assistance from the SCA.

Plan of investigation
Please comment on the quality and feasibility of the plan of investigation, its strengths and weaknesses, and its likelihood of success. Please highlight any areas of the plan that may require improvement.

The study is generally well described and is a rather straight-forward RCT. I agree with the applicants that individual level randomisation is feasible from the viewpoint of the SCAs. I do have residual concern over possible contamination between smokers. Some strategy might be put in place to ensure only one person from a household was included as it is not uncommon for multiple smokers in a home to try to quit together, and being in different arms could cause resentment. Asking about access to other forms of support at follow-up will be important and can be used to check for "contamination", something I like to think of as "diffusion".

The nature of the intervention remains a little obscure, perhaps inevitably given the limited space. Given that the SCA has some training in smoking cessation and will be providing some general advice,

it is unclear to me to what extent they are being encouraged to discuss the content of the 4 page tailored letter with the clients. Some provision for discussion should be allowed. This might occur at the initial visit, or some subsequent visit. SCAs will need to be trained in how to make maximum use of the intervention, which should include ensuring they have a sufficient understanding of the strategies embedded within it to discuss it meaningfully with clients, especially those who may have some problems in comprehension. That said, having the written guide can be of enormous help to such people in prompting them and as it requires less memory, of ensuring that the key information they need is available.

More consideration may be required regarding the 5 pound voucher for sending text messages to the system. This may end up being an important part of the intervention as it may motivate greater engagement with the texting part of the program. I think it should be included as an explicit part of the care package if it could be replicated in practice.

I think the basic idea of complementing face-to-face advice with providing of an automated form of help is likely to be the preferred approach of much cessation support in future. This is part of what makes this proposal so timely and important. There are vvery few studies of this in any setting. Smokers are reluctant to return for multiple visits to a face to face service, as it is expensive in terms of time for them, and most only need minimal prompts and some basic organisational support to progress, which the automated intervention is designed to provide.
In the economic analysis it will be impotant to see if the intervention results in more or fewer visits for assistance and of the duration of such visits. The intervention could reduce the need for additional face-to-face follow-ups as it provides information and support they might otherwise seek, but alternatively could concretise concerns and encourage more use of the face-to-face assistance.

Track record of Lead Applicant/research group
Please comment on the suitability of the Lead Applicant / research group for the proposed studies.

The team is led by an acknowledged international expert in the area, and contains the breadth of experience of understanding of both smoking cessation and primary care to be successful. A first rate team.

Support requested
Please comment on the appropriateness of the support requested. Does this request represent value for money?

The overall budget is very reasonable for a trial of this size, importance, and complexity. I wonder if it needs two full-time staff for 42 months for recruiting 2 to 3 practices a month (66 over around 30 months) plus all of the other tasks of running a RCT. I note the timeline implies recruiting practices and beginning recruitment from the first month of funding. This implies that all Ethics requirements and programming of the surveys and consent procedures will be operational and tested from day 1. I suggest some time be allocated for this and the actual period of recruiting smokers be squeezed up as a result. I also wonder if it is preferable to have the statistician and health economist on a fractional basis before the last 6 months. The economic analyses can largely start once recruitment is complete, and there are important analyses do on the baseline data that could take up some of the time the statistician. If this was done I am not sure they would need full-time work for the last 6 months. Overall, I suspect the research support (as distinct from the recruitment support) is a bit low, so perhaps the overall amount is about right.

Overall Assessment
Please provide an overall assessment of the application.

This is an extremely important proposal which the potential to transform the provision of smoking cessation help within primary care setttings in an extraordinarily cost-effective way. Integation of face-to-face and computer generated automated supports is likely the way of the future, and this project is thus leading the exploration of the likely impacts.

Cancer Research UK
Peer Review Comments

External Reviewer 2

This page will be fed back to the applicant intact; please do not include any reference to your identity, or any particularly sensitive comments.

Importance of the proposal and its suitability for funding by Cancer Research UK

Please comment on the importance of the proposed work, its likely impact in the field of cancer research, and its suitability for funding by Cancer Research UK.

Smoking cessation interventions are the most effective and cost-effective interventions to reduce the risk of cancer. The proposal aims to assess a smoking cessation intervention which is very cheap and which can be easily implemented in routine primary care. Therefore, although the relative effectiveness in helping smokers to quit long-term with this intervention may be low(as indicated in the sample size calculation; 5% difference in effect with usual care) the interventions' overall impact may still be high.

Originality of the work

Please comment on the novelty of the proposed studies. Have they been duplicated by others in the field?

The proposed nature of the intervention (tailored report and text messages) in itself is not new. However, the current work builds on previous work by the authors which is a strength with regard to assessing an intervention which is supposed to be implemented widely if proven to be effective.

Plan of investigation

Please comment on the quality and feasibility of the plan of investigation, its strengths and weaknesses, and its likelihood of success. Please highlight any areas of the plan that may require improvement.

Overall, the plan is written clearly and concisely. The proposed intervention is not new, but it is very relevant to prove the effectiveness of such a behavioural support program in a pragmatic trial. The internal validity of the proposed trial is high. A few concerns remain (see below).

Major comments

1. Background (page 1-2). There is no mention of the authors' earlier, big trial (ESCAPE) which failed to detect an effect of tailored materials (reference: Gilbert et al. Addiction 2013; 108:811-819). Furthermore, the authors argue that their pilot trial showed an effect of their iQuit in practice intervention at long-term. However, that pilot was designed to assess short-term effectiveness and failed to detect a difference. The authors state that their iQuit system is "highly tailored" but fail to provide evidence that such tailoring is indeed needed/ better than other text messaging interventions which are not tailored (such as, e.g., "txt2stop"). The current design cannot provide an answer to that question because the tailord iQuit system is compared with usual care. One might argue that the "txt2stop" intervention would be even more easily implemented than iQuit as it does not require tailoring and because it already exists. What arguments do the authors then have to spend resources on developing/testing another (their) system?

2. Procedure (page 4). The authors do not explain at what point in the trial informed consent will be sought. It should also be specified what information is given to participants in this regard prior to randomisation. These aspects are important for the internal validity of the trial.

<u>Minor comments</u>

3. This reviewer wonders whether the use of smartphone "apps" would have greater potential than "old school" text messages?
4. Recruitment: practices. Is recruiting 66 practices into the trial feasible?
5. Recruitment: participants. Suggest to specify "current *tobacco* smokers" (to distinguish from e-cig users). Also: should you exclude those who use other aids to cessation than medications as well (e.g., use of e-cigs)?
6. Interventions/control. Clarify if every participant receives a prescription for smoking cessation medication.
7. Procedure. How well can the generated report be understood by deprived (low-literate) smokers? Do the authors have any previous evidence on that?
8. Procecure. Please specify "resource use".
9. Analysis. It would be very interesting to also present outcomes by practice to prove homogeneity.
10. Milestones for sharing. Suggest to have the trial protocol published much earlier (within first year) to reduce the risk of changes to the original plan informed by any interim findings.

Track record of Lead Applicant/research group

Please comment on the suitability of the Lead Applicant / research group for the proposed studies.

The track record of the leading researchers is very good and the composition of the group as a whole is adequate.

Support requested

Please comment on the appropriateness of the support requested. Does this request represent value for money?

The requested support seems justified and represent value for money.

Overall Assessment

Please provide an overall assessment of the application.

The authors' proposal is clear and concise and describes the assessment of an intervention consisting of a tailored report plus text messages to improve the effectiveness of care as usual for smoking cessation. If they indeed prove the effectiveness of their intervention, this could have a population impact on helping smokers quit.

Cancer Research UK
Peer Review Comments

External Reviewer 3

This page will be fed back to the applicant intact; please do not include any reference to your identity, or any particularly sensitive comments.

Importance of the proposal and its suitability for funding by Cancer Research UK
Please comment on the importance of the proposed work, its likely impact in the field of cancer research, and its suitability for funding by Cancer Research UK.

Smoking is the leading modifiable risk factor for cancer, and smoking cessation is one of the most cost-effective interventions for improving health. The proposed intervention has the potential to prevent or reduce the risk of cancer in many thousands of people, and is therefore highly relevant to Cancer Research UK.

Originality of the work
Please comment on the novelty of the proposed studies. Have they been duplicated by others in the field?

The proposed trial involves the combination of two individually-proven smoking cessation interventions (text messaging and written advice) into a 'package of care' delivered through primary health care. The novelty of the study is around the combination of the two interventions and the tailoring of the advice.

Plan of investigation
Please comment on the quality and feasibility of the plan of investigation, its strengths and weaknesses, and its likelihood of success. Please highlight any areas of the plan that may require improvement.

The objectives and time frame seem realistic, and the team have undertaken the necessary preliminary work to support the trial design. The argument provided about whether the trial design should be cluster or not is well argued. My comments below are minor:

- Information on secondary outcomes is a little scarce: E.g. will the number of follow-up visits related to usual care be recorded and will participants in the intervention arm be asked how often (if ever) they referred to the written advice provided? Can you confirm that the only follow-up time points are at 4 weeks and 6 months? Are any additional data (other than abstinence and cost/utility) recorded at these time points? Doesn't the EQ-5D also need to be asked at baseline?

- What cut-off will be used for cotinine in the saliva samples to say a person is abstinent, and what cut-off will be used for CO?

- I realise that not everyone can be followed out to 12 months given the time and cost constraints of the trial. However, would it be possible to follow-up a subsample of people out to 12 months

(e.g. Those recruited in the first 6 months of the trial) and collect abstinence data? Longer term abstinence information would be of great interest.

Track record of Lead Applicant/research group

Please comment on the suitability of the Lead Applicant / research group for the proposed studies.

The study team appear highly-skilled and well-qualified to undertake this work within the desired time frame and within budget.

Support requested

Please comment on the appropriateness of the support requested. Does this request represent value for money?

As mentioned above, smoking cessation is one of the most cost-effective interventions for improving health. The budget requested appears well justified and valid. No mention of support for the CO monitors was made so I assume they already have them?

Overall Assessment

Please provide an overall assessment of the application.

This is an extremely well-written and well thought out trial and I can find little to fault it. The design is robust, the study team strong and the budget realistic.

IN CONFIDENCE
Cancer Research UK Peer Review Comments

External Reviewer 4

This page will be fed back to the applicant intact; please do not include any reference to your identity, or any particularly sensitive comments.

Importance of the proposal and its suitability for funding by Cancer Research UK
Please comment on the importance of the proposed work, its likely impact in the field of cancer research, and its suitability for funding by Cancer Research UK.

The proposal concerns the effectiveness and economic evaluation of IQuit in Practice, a smoking cessation intervention for general practices in which face-to-face support, a written tailored advice and a three months follow-up programme of automated tailored telephone text messages are combined. In a high quality RCT this intervention will be compared with usual care on 6 months smoking cessation- and economic outcomes. The proposal is well written by an excellent project group.
Strengths of the proposal include: the study design, the systematic plan of research, the systematically developed intervention and the inclusion of a cost effectiveness evaluation. I am also convinced that the research group will be excellent in performing this well designed study. As the project aims at optimalization of smoking cessation interventions and smoking cessation lowers the risk on smoking-related cancers the work will positively impact the field of cancer research.
What lowers the importance of the proposal is that it has only a little surplus value compared to the researchers' own pilot study (Naughton et al., 2014) which already showed the six months effectiveness and feasibility of the intervention. This pilot already compared the intervention with usual care in the same setting with 32 GPs and 602 patients. In fact a similar study is planned, though including an economic evaluation now (the researchers state in their proposal that they also already have some insight into the costs of the IQuit in Practice). The impact would be higher if more attention would be paid to implementation (see later comments).

Originality of the work
Please comment on the novelty of the proposed studies. Have they been duplicated by others in the field?

My main concern on the proposal is the value of the output given to what the researchers already know (from own preliminary studies) about the effectiveness of the intervention and given the large costs (1 research associate, 3 research assistants, and about 20h per week supervision (latter is not applied for in the financial grant, own resources).

Plan of investigation
Please comment on the quality and feasibility of the plan of investigation, its strengths and weaknesses, and its likelihood of success. Please highlight any areas of the plan that may require improvement.

The plan is well-written, complete and of high quality. However, I have remarks / considerations with regard to the choices the project group has made with regard to the main aims of the project (see previous comments). My main issues are: - To make the project more beneficial for practice and science I suggest that the RCT also measures 12 months effectiveness and cost effectiveness outcomes (is now only 6 months).

- Given the preliminary work of the researchers on this intervention, I would suggest to place more emphasis on implementation of the IQuit in Practice (e.g. how to manage/facilitate that all eligible smokers in GPs are approached by the SCA and that smokers are going to use and adhere to the programme etc.), and on strategies that facilitate the phases of the diffusion process. It is recommended to combine this with the monitoring of cost and effects. In that way the researchers get more insight into long term costs and effects but also invest in nationwide implementation and integration of the intervention in practice.
- A related issue is that I miss information on what the researchers have learned from the pilot project and what kind of improvements they propose for the new project.
- Another related issue is the number of smokers that is estimated to be included per GP, namely one per month which makes it impossible for GPs to get routine. - Although economic evaluation is one of the main aims, the proposal give very little information on the precise types (full economic evaluation?), content and how it is going to be performed. It would also be worthwhile to include a long term modelling study. Moreover QOL measurements (EQ5D) that are so far often used in smoking cessation economic evaluations are critized and need improvement. I miss a critical reflection on what so far has been known about cost effectiveness of smoking cessation interventions and what can be learned from them to optimize it in the proposed project.
- Although I understand that it may be necessary to include new GP practices for an optimal study design it might also be valuable to include these practices to find out whether effectiveness improves with the extent to which the protocol has become common practice and CSAs become more experienced in performing it.
- Given the rapid development of Smartphones and tablets I would also be interesting whether and how the intervention could be offered via these deliverables.

Track record of Lead Applicant/research group
Please comment on the suitability of the Lead Applicant / research group for the proposed studies.

The Lead applicant has an excellent track record in the area of smoking cessation, eHealth interventions and in conducting effectiveness evaluation studies (RCT’s). There is also a strong project group that adds necessary expertise in conducting cost-effectiveness studies and in implementing smoking cessation interventions in GPs. It is also a strength that a GP participates in the project group as the study concentrates on smoking cessation in the GP setting.

Support requested
Please comment on the appropriateness of the support requested. Does this request represent value for money?

The added value of the proposed research concerns the full economic evaluation and the biochemical validation as a preliminarily pilot RCT already showed the effectiveness and feasibility. The preliminary work makes that several products (intervention, protocols,

trainingtools, questionnaires etc) are available. This makes me doubt that the proposed personnel investment is needed (1 research associate, 3 research assistants, and about 20h per week supervision (latter is not applied for in the financial grant, own resources). Other costs seem realistic.

Overall Assessment
Please provide an overall assessment of the application.

I underline the great value of testing effectiveness and cost effectiveness of smoking cessation interventions in general and specifically in general practice and I put high value on the well designed RCT. My enthousiasm for this proposal is however tempered because I am not really convinced about its added value above what is already known on the (cost) effectiveness of this intervention (and comparable interventions) combined with the lttle attention implementation receives.
