# Supplementary material for: Assessment of the Effectiveness and Cost-Effectiveness of Tailored Web- and Text-Based Smoking Cessation Support in Primary Care (iQuit in Practice II): Protocol for a Randomized Controlled Trial
Source: JMIR Res Protoc. 2020 Jul 14;9(7):e17160. doi: 10.2196/17160 (PMC7388034; doi:10.2196/17160)
Supplement: Multimedia Appendix 9 [file resprot_v9i7e17160_app9.docx]

| **Baseline variables (t_0_)** | **Intervention** | **Control** |
| --- | --- | --- |
| **Online Questionnaire** | | |
| Age | X | X |
| Gender | X | X |
| No. Cigarettes smoked per day | X | X |
| Time first cigarette smoked after waking | X | X |
| Motivation to quit | X | X |
| Confidence to quit | X | X |
| When is your quit date? | X | X |
| CO reading | X | X |
| Type of cigarette smoked | X |  |
| Money spent on smoking per week | X |  |
| Number of previous quit attempts | X |  |
| How much do you agree that ‘I am able to see myself as a non-smoker?’ | X |  |
| Important reasons for quitting smoking | X |  |
| Situations in which it might be difficult to resist smoking | X |  |
| Possible advantages of quitting | X |  |
| Possible disadvantages of quitting | X |  |
| No. adults living in household | X |  |
| Do any of them smoke? | X |  |
| Do any of them plan to change their smoking habits? | X |  |
| No. of children under the age of 16 | X |  |
| How supportive are others to help you quit? | X |  |
| Do you have any health problems linked to smoking? | X |  |
| **EQ5D-5L questionnaire** | | |
| Any mobility problems | X | X |
| Any self-care problems | X | X |
| Any problems with usual activities | X | X |
| Any problems with pain or discomfort | X | X |
| Any anxiety or depression | X | X |
| Self-report of current health | X | X |
| **Other variables** | | |
| Occupation (from GP records) | X | X |
| Ethnicity (From GP records) | X | X |
| Postcode (From Consent Form) | X | X |
