# Supplementary material for: Assessment of the Effectiveness and Cost-Effectiveness of Tailored Web- and Text-Based Smoking Cessation Support in Primary Care (iQuit in Practice II): Protocol for a Randomized Controlled Trial
Source: JMIR Res Protoc. 2020 Jul 14;9(7):e17160. doi: 10.2196/17160 (PMC7388034; doi:10.2196/17160)
Supplement: Multimedia Appendix 10 [file resprot_v9i7e17160_app10.docx]

| **Outcome Variables** | **Time point** | **Intervention** | **Control** |
| --- | --- | --- | --- |
| Carbon monoxide reading at 4-weeks post quit date  (collected from the SCA) | t_2_ | X | X |
| **6-month follow-up questionnaire** | | | |
| Have you smoked since your initial quit date 6 months ago  (Collected via text where possible) | t_4_ | X | X |
| Have you smoked in the last 7 days? | t_4_ | X | X |
| No. quit attempts over the 6-month study period? | t_4_ | X | X |
| Motivation to quit for good | t_4_ | X | X |
| Confidence to quit for good | t_4_ | X | X |
| Use of Medications and nicotine products over the 6 month study period | t_4_ | X | X |
| How long medications and nicotine products used | t_4_ | X | X |
| Advice or support from other professionals to help stop smoking during the 6 month study period | t_4_ | X | X |
| Tactics and how many times used to help avoid smoking during the 6-month study | t_4_ | X | X |
| Any mobility problems | t_4_ | X | X |
| Any self-care problems | t_4_ | X | X |
| Any problems with usual activities | t_4_ | X | X |
| Any problems with pain or discomfort | t_4_ | X | X |
| Any anxiety or depression | t_4_ | X | X |
| Self-report of current health | t_4_ | X | X |
| Did you read the advice report? | t_4_ | X |  |
| Did you read the text messages | t_4_ | X |  |
| How helpful did you find the advice report? | t_4_ | X |  |
| How helpful did you find the text messages? | t_4_ | X |  |
| How did you find the text messages | t_4_ | X |  |
| How did you feel about the number of texts sent? | t_4_ | X |  |
| What did you think of the duration of the text messaging program overall | t_4_ | X |  |
